# *Plasmodiophora brassicae*-Triggered Cell Enlargement and Loss of Cellular Integrity in Root Systems Are Mediated by Pectin Demethylation

**DOI:** 10.3389/fpls.2021.711838

**Published:** 2021-07-29

**Authors:** Karolina Stefanowicz, Monika Szymanska-Chargot, William Truman, Piotr Walerowski, Marcin Olszak, Adam Augustyniak, Arkadiusz Kosmala, Artur Zdunek, Robert Malinowski

**Affiliations:** ^1^Institute of Plant Genetics, Polish Academy of Sciences, Poznan, Poland; ^2^Institute of Agrophysics, Polish Academy of Sciences, Lublin, Poland; ^3^Institute of Biochemistry and Biophysics, Polish Academy of Sciences, Warsaw, Poland; ^4^Centre for Advanced Technology, Adam Mickiewicz University, Poznan, Poland

**Keywords:** clubroot disease, *Plasmodiophora brassicae*, cell enlargement, cell wall, *Arabidopsis thaliana*

## Abstract

Gall formation on the belowground parts of plants infected with *Plasmodiophora brassicae* is the result of extensive host cellular reprogramming. The development of these structures is a consequence of increased cell proliferation followed by massive enlargement of cells colonized with the pathogen. Drastic changes in cellular growth patterns create local deformities in the roots and hypocotyl giving rise to mechanical tensions within the tissue of these organs. Host cell wall extensibility and recomposition accompany the growth of the gall and influence pathogen spread and also pathogen life cycle progression. Demethylation of pectin within the extracellular matrix may play an important role in *P. brassicae*-driven hypertrophy of host underground organs. Through proteomic analysis of the cell wall, we identified proteins accumulating in the galls developing on the underground parts of *Arabidopsis thaliana* plants infected with *P. brassicae*. One of the key proteins identified was the pectin methylesterase (PME18); we further characterized its expression and conducted functional and anatomic studies in the knockout mutant and used Raman spectroscopy to study the status of pectin in *P. brassicae*-infected galls. We found that late stages of gall formation are accompanied with increased levels of PME18. We have also shown that the massive enlargement of cells colonized with *P. brassicae* coincides with decreases in pectin methylation. In *pme18-2* knockout mutants, *P. brassicae* could still induce demethylation; however, the galls in this line were smaller and cellular expansion was less pronounced. Alteration in pectin demethylation in the host resulted in changes in pathogen distribution and slowed down disease progression. To conclude, *P. brassicae*-driven host organ hypertrophy observed during clubroot disease is accompanied by pectin demethylation in the extracellular matrix. The pathogen hijacks endogenous host mechanisms involved in cell wall loosening to create an optimal cellular environment for completion of its life cycle and eventual release of resting spores facilitated by degradation of demethylated pectin polymers.

## Introduction

Clubroot disease, caused by the protist pathogen *Plasmodiophora brassicae*, is responsible for extensive damage to oilseed rape and brassica vegetable crops. An obligate biotroph, *P. brassicae*, grows intracellularly in host root systems and manipulates the development of the belowground parts of the plant to form distinctive galls. Cellular reprograming in the underground parts of plants allows the pathogen to acquire host nutrients and secure space for resting spore formation (Malinowski et al., 2019). Eventually, root systems of infected plants disintegrate and resting spores are released to soil where they retain their infection potential for up to 20 years. Enormous growth of pathogen-colonized cells and local cell wall decomposition that accompanies the emergence of secondary plasmodia, followed by resting spore formation and maturation, is a characteristic symptom of the clubroot disease (Gustafsson et al., 1986). It has been shown that this cellular enlargement is mediated by brassinosteroids (Schuller et al., 2014) and involves both cell wall remodeling (Devos et al., 2006) and endoreduplication (Olszak et al., 2019). Schuller et al. (2014) proposed that this response may be mediated by auxin-brassinosteroid crosstalk, disturbance of which leads to changes in pathogen distribution and development. The induction of host plant organ hypertrophy is a common strategy of pests and pathogens, used to establish beneficial trophic relations and facilitate propagation of the parasite. In nature, we can observe numerous examples, for example, the leaf rolling caused by galling insects or galls induced by *Agrobacterium tumefaciens* as well as galls on roots generated by nematodes or *P. brassicae* (Gätjens-Boniche, 2019). Host organ hypertrophy provides space for the pathogen and elevates cellular metabolism, redirecting nutrient distribution and source sink relations within the plant (Chandran et al., 2010; Bragança et al., 2016; Walerowski et al., 2018). Cell wall changes not only are essential for the expansion and enlargement but also contribute to the restriction of pathogen spread within the host (Bohlmann and Sobczak, 2014). The capacity of pathogens to enzymatically degrade cell walls of the host also influences their infectivity and release back to the environment (Walton, 1994).

Turgor-driven plant cell growth requires constant adjustment of cell wall composition – its synthesis, loosening and subsequent reinforcement (Wei and Lintilhac, 2007). These events have a vast impact on tensions at the organ level; therefore, regulation of cell wall dynamics involves appropriate control of the cell adhesion/cell separation balance that is highly dependent on pectin status, and secondary cell wall modifications (Jarvis et al., 2003). Transcriptomic and proteomic studies of clubroot disease have identified extensive changes in host genes and proteins involved in cell wall synthesis and turnover (Devos et al., 2006; Badstöber et al., 2020). Additionally, host cell wall degradation or thickening has been documented at the microscopic level (Donald et al., 2008). So far, however, only the action of xyloglucan endotransglucosylase/hydrolase (XTH) proteins on the primary cell wall during gall growth has been functionally characterized (Devos et al., 2005). In this study, we investigated changes in proteins associated with the cell wall at two time points: 20 days after infection (DAI) when pathogen-driven cell expansion is occurring and 26 DAI when both cell enlargement, and the cell degradation related to pathogen spore release is taking place. Among the differentially accumulating proteins, we found Pectin Methylesterase 18 (PME18) and followed up functionally characterizing its potential role in facilitating *P. brassicae*-driven organ hypertrophy. Furthermore, we used Raman spectroscopy to profile the pectin status of cell walls enclosing pathogen-colonized, enlarged cells. We discuss the importance of pectin demethylation for the course of later stages of *P. brassicae*-induced gall development.

## Materials and Methods

### Plant Material

In all experiments, *Arabidopsis thaliana* accession Columbia-0 (Col-0) was used as a control. The mutant *pme18-2* T-DNA single insertion line (SALK_076975; Alonso et al., 2003) in Col-0 background was obtained from the Nottingham Arabidopsis Stock Centre^1^ (ID N576975) as single, segregating flank-tagged T3 generation. Homozygous mutant plants were selected on 0.6% (w/v) agar medium (BioShop) containing Murashige and Skoog salts (MS; Duchefa) and 75 mg L^−1^ kanamycin according to the protocol by Harrison et al. (2006) and were tested for T-DNA insertion and homozygosity with PCR using primers designed by T-DNA Primer Design Tool^2^ (Supplementary Table S1). During the selection process, we observed phenotypic segregation where only plants homozygous for the T-DNA insertion had altered rosette size. Selected homozygote mutant plants were also tested for the presence of the *PME18* gene transcript, and only residual levels were detected (Supplementary Figure S1). For this, relative gene expression was calculated and normalized with REST-MCS software using three reference genes (*PP2A-At1g13320, TIP41-At4g34270* and *UBC9-At4g27960*; Supplementary Table S1) according to Pfaffl et al. (2002).

### Growth Conditions and Inoculation

All experiments were carried out under controlled growth conditions at a light irradiance of 100 μmol m^−2^ s^−1^, with a 9 h light/15 h dark photoperiod and temperatures of 22°C day/20°C night and 65% humidity. Seeds were surface sterilized by immersion for 2 min in 70% ethanol, followed by 10 min in 5% sodium hypochlorite (commercial bleach), washed in sterile water, kept for 5 days at 4°C and germinated *in vitro* on half-strength MS agar plates containing 0.6% (w/v) agar and 1% (w/v) sucrose, pH 5.7. 10 days after germination, selected seedlings (with rosette size ranging from 1.2 to 1.5 cm) were transferred to soil substrate (Kronen-Klasmann Potgrond LT 011 and perlite, 7:1). 17 days after germination, plants were infected with 2 ml of *P. brassicae* P1 (Some et al., 1996) spore suspension at a concentration of 1 × 10^6^ spores ml^−1^. Inoculum was prepared as described by Malinowski et al. (2012). Control plants were mock-treated with 2 ml of water. For cell wall protein extractions, hypocotyl tissue was collected 20 and 26 days after inoculation (DAI) in two independent biological replicates, each with 90 plants per combination. For RNA extractions, hypocotyl tissue was collected 7, 10, 16, 20 and 26 DAI in three independent biological replicates, each with 30 plants per combination. Collected hypocotyl samples were immediately frozen in liquid nitrogen and stored at −70°C until further processing. For microscopy, hypocotyl tissue was collected 20 and 26 DAI and processed as described further.

### Cell Wall Isolation and Protein Extraction

Purification of cell walls from the hypocotyl tissue and cell wall protein extraction were performed according to the method described by Feiz et al. (2006) with some modifications. For each sample, equal amounts of frozen hypocotyl tissue were homogenized in a mortar using low ionic strength 5 mm acetate buffer (pH 4.6) containing 0.4 M sucrose and protease inhibitor cocktail (cOmplete Mini EDTA-free, Sigma-Aldrich). After adding polyvinylpyrrolidone (PVP; 1 g per 10 g fresh weight of hypocotyls), the homogenate was incubated for 30 min on ice with stirring on a rotary platform. Cell walls were separated from cytoplasmic content by centrifugation at 1000 × *g* for 15 min at 4°C. The pellet was then purified by two successive centrifugations at 1000 × *g* for 15 min at 4°C in 5 mm acetate buffer (pH 4.6), containing 0.6 M and 1 M sucrose, respectively. The remaining pellet was washed with 5 mm acetate buffer (pH 4.6) on a 40 μm cell strainer (Corning). The resulting cell wall fraction was ground to a fine powder in liquid nitrogen using a mortar. Proteins were extracted by vortexing of the cell wall powder for 10 min at room temperature in 0.2 M CaCl_2_ solution in 5 mm acetate buffer (pH 4.6) containing protease inhibitor cocktail, followed by centrifugation at 4000 × *g* for 15 min at 4°C. The supernatant was collected, and the extraction procedure was repeated on the remaining pellet. The two supernatants were pooled, and the obtained cell wall protein extract was desalted and concentrated on Amicon Ultra-0.5 ml centrifugal filters with 3 kDa mW cut-off (Millipore).

### Two-Dimensional Gel Electrophoresis (2D GE) and Protein Identification

Protein concentration of the cell wall protein extracts was estimated using the 2D Quant Kit (GE Healthcare, Buckinghamshire, United Kingdom). Proteomic analysis of the complete cell wall protein extracts from 3 g of hypocotyl tissue (200–250 μg of protein per sample), including 2D GE and mass spectrometry (MS), was performed as described by Perlikowski et al. (2016) with some modifications. In the first dimension, isoelectric focusing (IEF), 24 cm dried gel strips (IPG *Blue*Strips, Serva) with immobilized linear pH range 6–10 were used to focus extracted proteins. Rehydration and focusing were performed in an Ettan IPGphor II (GE Healthcare) at 20°C at 50 μA per strip, with the following program: 12 h of rehydration at 0 V and 9 h of focusing (1 h at 500 V, 2 h at 1000 V, and 6 h at 8000 V). In the second dimension, the proteins were separated by SDS-PAGE, using 13% polyacrylamide gels (1.5 × 255 × 196 mm). The gels were stained with colloidal Coomassie Brilliant Blue (BioShop) G-250 using the modified method of Neuhoff et al. (1988), scanned with an ImageScanner III (GE Healthcare) and processed with LabScan 6.0 program (GE Healthcare). Spot detection and image analysis (spot matching) were performed using Image Master 2-D Platinum 6.0 software (GE Healthcare). The 2D GE analysis was performed for two independent biological replicates. Differential spots which were present only for the infected samples were excised from the gels and analyzed by liquid chromatography coupled with mass spectrometry at the Laboratory of Mass Spectrometry, Institute of Biochemistry and Biophysics, Polish Academy of Sciences (Warsaw, Poland). The raw data were analyzed with Mascot Distiller software (version 2.3, MatrixScience). The obtained peptide masses and fragmentation spectra were matched to the National Center Biotechnology Information (NCBI) non-redundant database with an Arabidopsis filter using the Mascot search engine (Mascot Daemon v2.3, Mascot Server v2.3.02; MatrixScience). Candidate matches with highest Mascot multidimensional protein identification technology score were chosen for further analysis. For unification of selected protein identities, additional BLAST-P comparison has been performed. The mass spectrometry proteomics data have been deposited to the ProteomeXchange Consortium *via* the PRIDE (Perez-Riverol et al., 2019) partner repository with the data set identifier PXD026660 and 10.6019/PXD026660.

### SDS-PAGE and Western Blot Analysis

Protein concentration of the cell wall protein extracts from 1 g of hypocotyl tissue was estimated with Qubit Protein Assay Kit using a Qubit 3 Fluorometer (Thermo Fisher Scientific). Extracellular samples containing 10 μg of protein were incubated with 5 × SDS sample buffer for 10 min at 95°C, resolved by SDS-PAGE under reducing conditions and electroblotted to 0.2 μm PVDF membranes (Immun-Blot PVDF, Bio-Rad). Blots were blocked with 5% (w/v) skim milk powder in Tris-buffered saline (TBS; 150 mm NaCl, 10 mm Tris, 0.1% (v/v) Triton X-100, pH 7.6) and incubated for 2 h with a 1:1000 diluted polyclonal rabbit anti-PME18 antibody conjugated to horseradish peroxidase (Bioss Antibodies, United States). Detection was performed using a colorimetric assay with 3,3′-diaminobenzidine tetrahydrochloride (Roth) as a substrate. Western blotting was performed for two independent biological replicates with 90 plants per combination, each with three technical replicates. Blots were scanned using a flatbed scanner (Hewlett-Packard Color LaserJet Pro M177), and protein quantity was estimated with ImageJ software (Schneider et al., 2012) by measuring pixel intensity (mean gray value) of the detected signal. Protein loading was visualized by membrane staining with 0.5% (w/v) Ponceau S in 1% acetic acid.

### Real-Time qPCR

RNA from hypocotyls was isolated with Total RNA Mini Plus Kit (A&A) according to manufacturer’s protocol, treated with DNase I (Thermo Fisher Scientific) and quantified using Nano-Drop equipment (Thermo Fisher Scientific). First-strand cDNA synthesis was performed on 2 μg of RNA with the RevertAid H Minus First Strand cDNA Synthesis Kit (Thermo Fisher Scientific) following manufacturer’s instructions. The RT-qPCR reactions were performed using the LightCycler 480 instrument (Roche) and the SensiMix SYBR No-ROX Kit (Bioline). Each amplification was performed using gene-specific oligonucleotide primers designed using the Primer3Plus tool for qPCR^3^ (Supplementary Table S1), and final cDNA template concentration in the reaction was 2 ng μl^−1^. The program was as follows: 10 min 95°C – 40 × (20 s 95°C – 20 s 60°C – 20 s 72°C) ending with a melting curve generation. Three technical replicates were combined to give an average value for each biological replicate, and three independent biological replicates were analyzed for each condition. Relative gene expression levels were calculated and normalized with REST-MCS software using the geometric mean of two reference genes (*TIP41-At4g34270* and *UBC9-At4g27960*; Supplementary Table S1) according to the method described by Pfaffl et al. (2002).

### Transcript Sequencing

The RNA-seq data analyzed here have been previously published by Malinowski et al. (2016), and the raw data from this experiment are available at the European Nucleotide Archive^4^ under the identifier PRJEB12261. Significantly differentially expressed genes were identified using the DESeq2 package in the R statistical environment with false discovery rates calculated using the *q*-value package (Love et al., 2014). Heat maps were generated using the pheatmap package in R.

### Light Microscopy

For anatomical observation and measurements, collected hypocotyls were incubated overnight at 4°C in PFA-GA fixative solution (2% paraformaldehyde, 2% glutaraldehyde, 1% caffeine, 1 × phosphate-buffered saline, and pH 7.4), dehydrated in a series of ethanol solutions of increasing concentrations (from 30 to 100%), embedded in Technovit 7100 resin (Kulzer) and cut on a rotary microtome (Leica). The obtained 5 μm sections were stained with 0.05% toluidine blue (Roth) solution (in citric phosphate buffer, pH 4.0), or with 0.3% alcian blue (Roth) solution (in 3% acetic acid) where safranin O (Acros Organics) was used as a counterstain. Alcian blue/safranin O double-stained sections were used for the quantification of de-esterified pectin with ImageJ software, where alcian blue staining area was measured using adjustment of threshold color in RGB color space and was calculated relative to the total area of hypocotyl section. Toluidine blue-stained sections were used for the measurements of hypocotyl width, enlarged cell size and number as well as hypocotyl expansion using ImageJ software. All sections were mounted in 50% glycerol solution and photographed using the Carl Zeiss AXIO Imager.M2 microscope system.

### Raman Spectroscopy

For Raman microspectroscopy, collected hypocotyls were embedded in Shandon Cryomatrix resin (Thermo Scientific), frozen in liquid nitrogen and stored at −70°C. Embedded, frozen tissue blocks were trimmed and cross-sectioned (30 μm thick) with the CM1520 cryomicrotome (Leica). The sections were attached to a microscope slide covered with aluminium foil to avoid the influence of glass bands on Raman spectra (Chylińska et al., 2014) and were dried at 60°C on a heating plate. The region of parenchymatic tissue was chosen for Raman imaging. For infected samples, the regions of enlarged, pathogen-colonized cells (large) and normal (small) cells were monitored separately. The Raman maps were obtained with a DXR Raman microscope (Thermo Scientific Inc., United States), equipped with diode-pumped, solid-state green laser *λ* = 532 nm with maximum power of 10.0 mW. The spectral resolution of the microscope was equal to 4 cm^−1^ with diffraction grating of 900 lines per mm and pinhole aperture of 50 μm. The system uses an air-cooled CCD detector, and the 20×/0.40 objective lens was used. Maps were recorded with spatial resolution of 2 μm in both x and y directions; the z direction was fixed during the map recording. The integration time was equal to 2 s and fixed for each scan. For each sample and region (normal and colonized cells), at least three maps were obtained. A single spectrum at each point was recorded in the range of 3500–150 cm^−1^ of Raman shift for an average of 20 scans. Image processing was performed in Omnic (Thermo Scientific, Waltham, MA, United States), and the spectra were not normalized. Raman chemical images for chosen bands were analyzed by Raman band integration. The band with maximum at 2936 cm^−1^, representing C-H stretching in cell wall polysaccharides, was integrated for visualization of whole plant cell wall. The degree of methylation of pectic polysaccharides in cell walls was evaluated based on position and intensity of peaks in the range between 840 and 857 cm^−1^ that represent stretching vibration of (1,4)-α-glycosidic bond in pectin molecules (Synytsya et al., 2003). All spectral processing was carried out using Origin Pro 8.5 (OriginLab Corporation, United States).

## Results

### Cell Wall Proteomics Reveals Increased Accumulation of Proteins Potentially Involved in Cell Enlargement or Tissue Degradation in Developing Clubroot Galls

**Figure 1:**
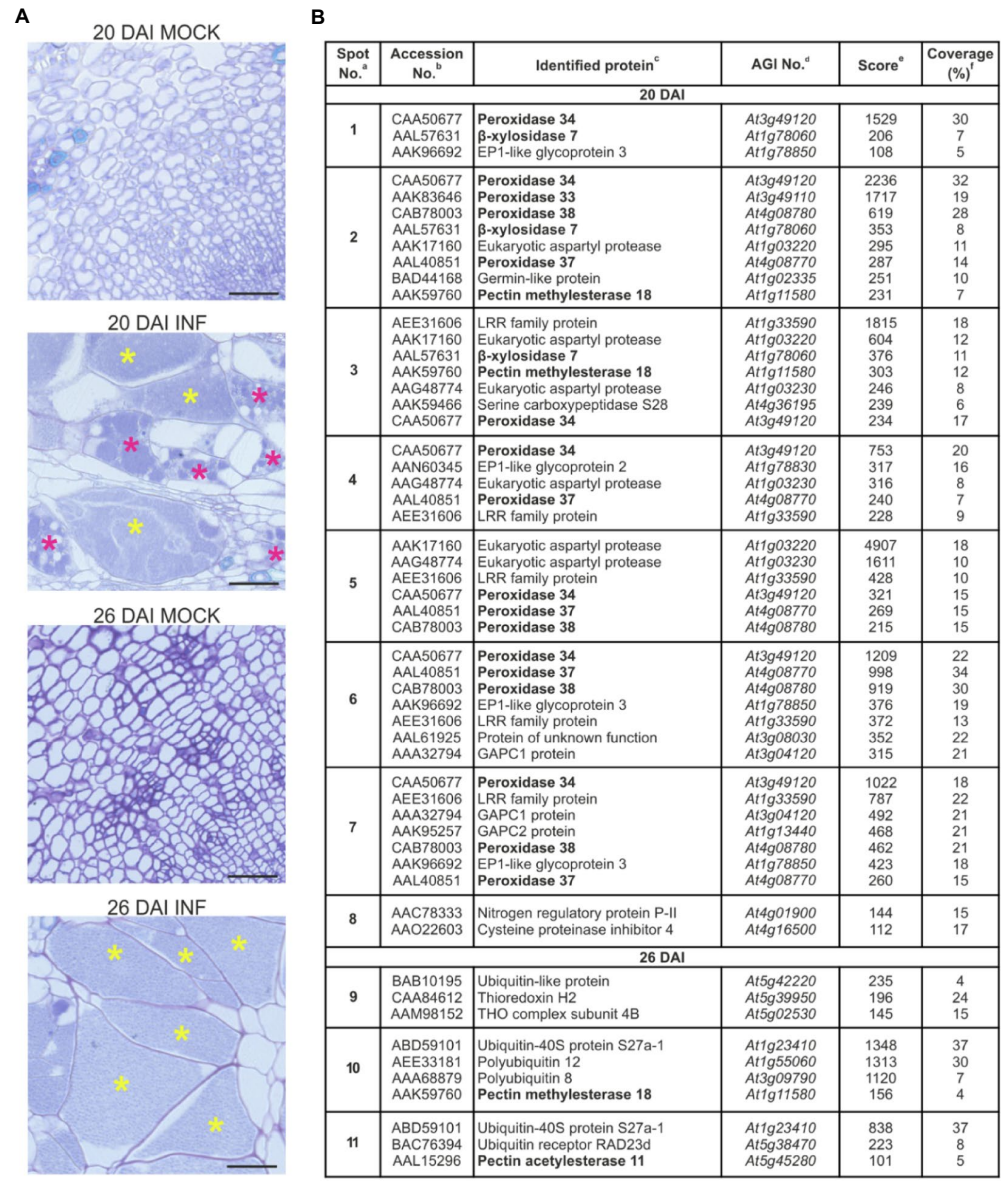
Proteomic analysis of the cell wall samples of Col-0 hypocotyls after infection with *Plasmodiophora brassicae*. **(A)** Toluidine blue-stained transverse 5 μm sections across hypocotyls of Col-0 plants before (mock) and after infection with *P. brassicae*, showing giant cell formation 20 DAI and 26 DAI. Scale bars represent 50 μm. Asterisks indicate enlarged cells filled with resting spores (yellow) or secondary plasmodia (magenta). **(B)** List of identified proteins (P-BLAST matching) together with images of differential protein spots (present only in samples isolated from infected plants) 20 DAI (spots marked 1–8) and 26 DAI (spots marked 9–11). Complete raw 2-D protein maps are shown in Supplementary Figure S1. Identified proteins potentially involved in cell wall remodeling are in bold. ^a^Spot numbering is the same as in panel **(B)**. ^b^Database accession (according to NCBInr) of a homologous protein. ^c^Homologous protein in Arabidopsis. ^d^Arabidopsis AGI No. ^e^Mascot multidimensional protein identification technology score. ^f^Amino acid sequence coverage for the identified proteins. Position of identified peptides mapped to full sequences is shown in Supplementary Figure S3.

Accumulation of extracellular proteins during the expansive growth of galls in *P. brassicae*-infected Arabidopsis hypocotyls was investigated by two-dimensional gel electrophoresis (2D GE). Protein samples isolated from cell walls of non-infected and infected (20 DAI and 26 DAI) samples were subjected to isoelectric focusing on pH gradient 6–10 and then resolution by SDS-PAGE. The first time point, 20 DAI, represents the transition phase between the proliferative stage of infection when the cells within infected hypocotyls are dividing and the expansive stage of infection when hypocotyl cells undergo severe hypertrophy. The second time point, 26 DAI, is an advanced stage of gall expansion when resting spore formation may be observed (Figure 1A). Analysis of the 2D gels revealed the presence of distinct differential protein spots specific for samples isolated from the infected plants at 20 DAI and 26 DAI (Supplementary Figure S2). Protein spots were further subjected to MS identification. All proteins identified (Figure 1B and Supplementary Figure S3) were encoded by the Arabidopsis genome, and no *P. brassicae* proteins were found. Most of the proteins detected at 20 DAI had extracellular localisation, and only a few of them were intracellular (e.g., serine carboxypeptidase S28 family protein and lactate/malate dehydrogenase family protein in spot No. 3, histidinol dehydrogenase in spot No. 5 or glyceraldehyde-3-phosphate dehydrogenase 1 and 2 in spots No. 6 and 7). In contrast, protein identification at 26 DAI returned primarily ubiquitin-related proteins which are characterized by nuclear and/or cytoplasmic localisation and as such should be considered as intracellular contamination. At this stage, galls contain numerous spore-filled enlarged cells and some of the host cells at an advanced stage of colonization have begun to disintegrate.

Seven of the proteins identified are potentially involved in cell wall remodeling, and these are marked in bold in Figure 1B. Largely these proteins belong to the peroxidase family, namely, PRX32, PRX33, PRX34, PRX37 and PRX38. These factors are typically involved in defense responses; however, they also play a role in cell expansion *via* ROS-mediated degradation of cell wall polysaccharides (Liszkay et al., 2003; Müller et al., 2009). Other spots containing proteins potentially involved in cell wall remodeling, namely, β-glycosidases BXL7 and BXL4, are known to be involved in the metabolism of arabinans, xylans and arabinoxylans and influencing cell wall flexibility (Minic et al., 2004; Gaudet et al., 2011). We also found increased accumulation of PME18 in response to infection. Pectin methylesterases (PME) potentially modify cell walls *via* the demethylesterification of pectin (Jolie et al., 2010). PMEs regulate the mechanical strength of the cell wall and are responsible for cellular adhesion (Daher and Braybrook, 2015). Previously, PME18 has been shown to contribute to immunity against the bacterial pathogen *Pseudomonas syringae* (Bethke et al., 2014), and so far, the role of this particular PME member in cellular growth has not been studied. In spot No. 4, we also identified peptides matching to heparanase-like protein 1. This β-glucuronidase activity-containing protein presumably changes the carbohydrate composition of the complex polysaccharide chains of arabinogalactan proteins at the cell surface and thereby affects the process of cell elongation (Eudes et al., 2008). One peptide identified in spot No. 11 matched to Pectin Acetylesterase 11, which potentially alters physical properties of the cell wall *via* pectin deacetylation (de Souza et al., 2014).

### Pectin Methylesterase 18 Protein and Transcript Accumulation Pattern Following Infection

**Figure 2:**
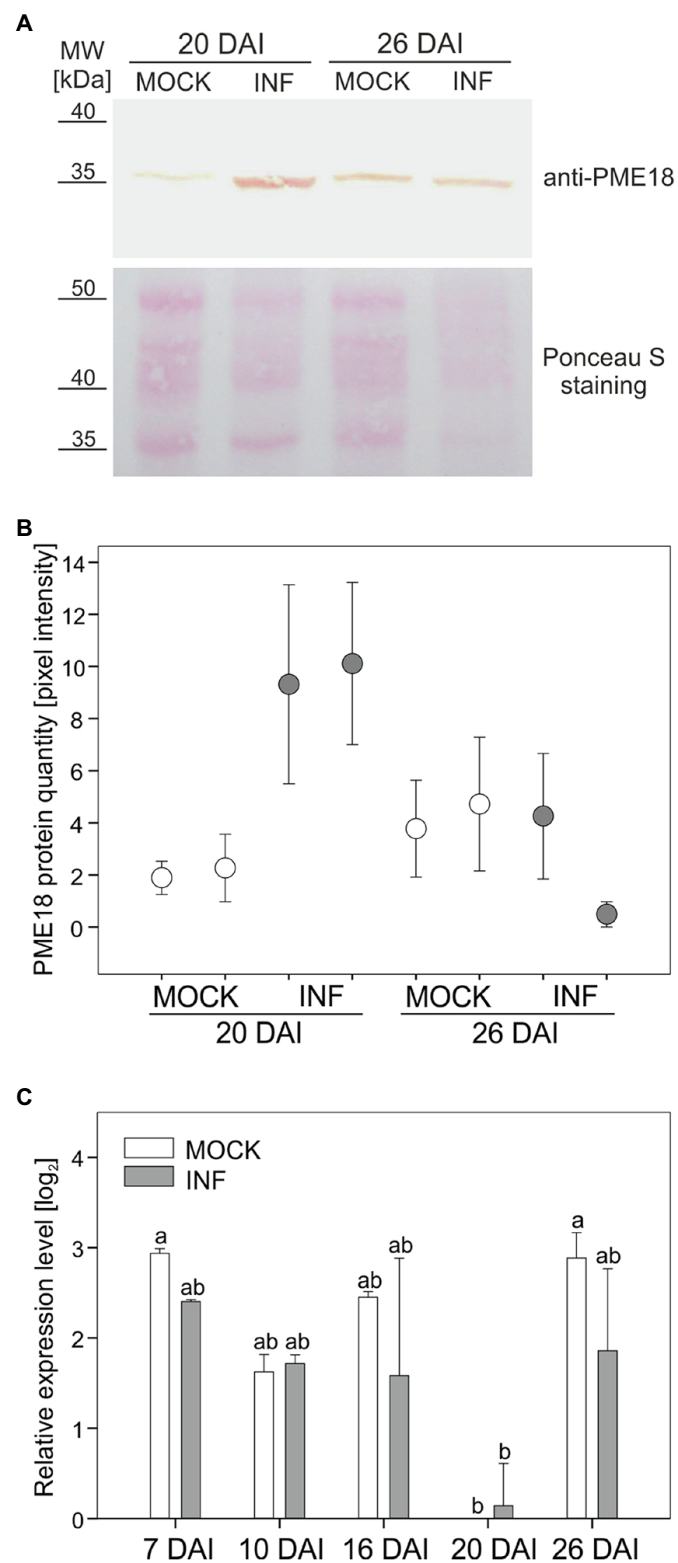
Pectin methylesterase (PME18) protein is differentially accumulated after infection with *P. brassicae*. **(A)** Western blot assay showing PME18 protein levels in the cell wall fraction from hypocotyls of mock-treated and *P. brassicae*-infected plants 20 DAI and 26 DAI. Total protein loading (10 μg) is depicted with Ponceau S PVDF membrane staining. **(B)** PME18 protein quantity measured as pixel intensity (mean gray value) of the Western blot bands. For each combination, two independent biological experiments (90 plants per sample) are plotted as two separate data points representing the mean of three technical replicates ± SD. **(C)** qRT-PCR analysis of *PME18* gene expression in the roots of mock-inoculated and *P. brassicae*-infected Col-0 Arabidopsis plants at indicated times (DAI). Transcript levels were normalized to two reference genes (*TIP41* and *UBC9*), calculated relative to the expression in mock-treated plants at 20 DAI and plotted as the means of three independent biological experiments (each with 30 plants per treatment) ± SE. Different letters indicate a significant difference between means (Tukey’s test, *p* < 0.05).

In this study, we decided to address in detail the potential role of PME18 accumulation in affecting the pectin status changes occurring during late stages of *P. brassicae* infection. To verify the proteomic findings, we made a quantitative analysis of PME18 protein accumulation in Col-0 plants before and after infection using the Western blot (WB) technique. This study only partially confirmed the validity of the proteomic results, since we observed an increased accumulation of PME18 in developing galls only 20 DAI and no statistical difference at 26 DAI (Figures 2A,B). Since the location of identified spots containing PME 18 on 2-D gels differs between 20 DAI and 26DAI (Supplementary Figure S2), we attribute observed difference between proteomic and WB studies to degradation processes that occurs during late stages of clubroot disease. We have not detected 62 kDa pre-processed PME band in the examined extracellular fraction. We followed our studies with RT-qPCR analysis and found that the PME18 protein accumulation is not reflected at the transcriptional level of *PME18* (locus *At1g11580*) since no statistically significant changes in comparable time points relative to non-infected controls were observed (Figure 2C). To obtain a broader view of the context of host transcriptional responses potentially related to cell wall remodeling and turnover that occurs in galls, we analyzed transcriptional data obtained for roots and hypocotyls at 16 and 26 DAI from our previous work (Malinowski et al., 2016). We found that despite the intense cellular growth within galls, members of the gene families related to cell wall remodeling or loosening do not always respond with transcriptional activation (Supplementary Figure S4). In developing galls at 26 DAI (hypocotyl region – the main site of gall formation in Arabidopsis) expression of only two cellulases, two pectate lyase and four PME genes increased significantly (Supplementary Figure S4). In the case of expansions, seven members were upregulated and 11 downregulated (Supplementary Figure S4). This situation underlines the importance of biochemical studies and proteomic studies for understanding cell wall status.

### *Plasmodiophora brassicae*-Driven Organ Hypertrophy Is Reduced in the *pme18-2* Mutant

**Figure 3:**
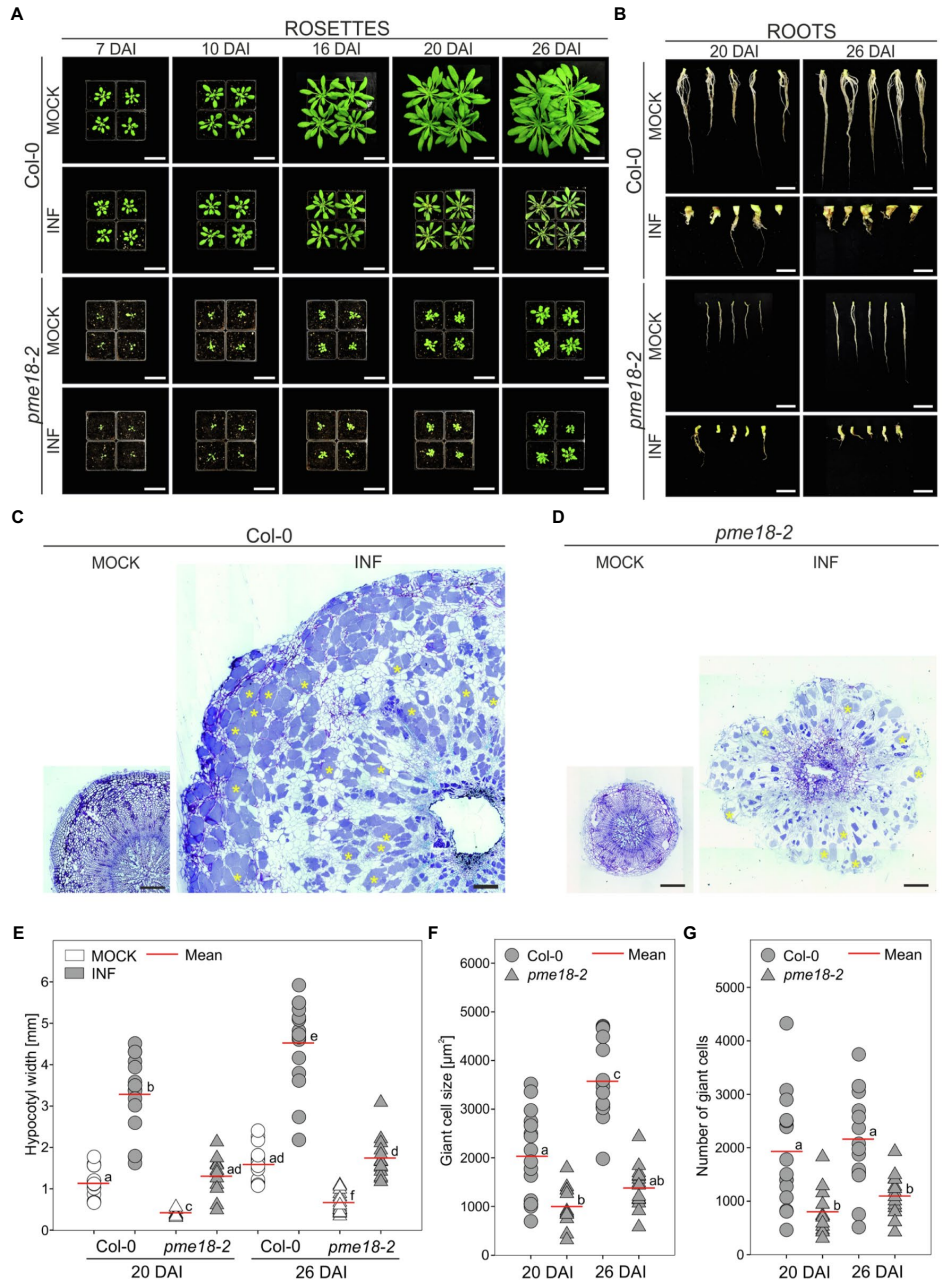
Clubroot disease development in wild-type Col-0 and *pme18-2*. The effects of disease progression on the morphology of aerial parts (rosettes) 7, 10, 16, 20 and 26 DAI **(A)**, and roots 20 and 26 DAI **(B)**. Scale bars represent 5 cm for rosettes and 1 cm for roots. **(C,D)** Representative transverse 5 μm sections across hypocotyls of Col-0 **(C)** and *pme18-2* **(D)** plants 26 DAI. Exemplary cells filled with resting spores are indicated with yellow asterisks. Sections were stained using toluidine blue. Scale bars represent 200 μm. **(E)** Hypocotyl width in mock- and *P. brassicae*-inoculated Col-0 and *pme18-2* plants 20 DAI and 26 DAI. Scatter plots present individual hypocotyl width measurements (between seven and 17 replicates for each combination), calculated means and SEs. Different letters indicate significant differences between means (Tamhane’s test, *p* < 0.05). **(F)** The influence of the *pme18-2* mutation on the size of enlarged cells 20 DAI and 26 DAI. Scatter plots present individual measurements performed on radial sections of 7 to 17 independent hypocotyls for each combination, calculated means and SEs. Different letters indicate significant differences between means (Tamhane’s test, *p* < 0.05). **(G)** The influence of the *pme18-2* mutation on the number of giant cells 20 DAI and 26 DAI. Scatter plots present individual calculations of enlarged cells for 7 to 17 radial sections taken from independent hypocotyls for each combination, calculated means and SEs. Different letters indicate significant differences between means (Tamhane’s test, *p* < 0.05).

Since we did not observe differential accumulation of PME proteins other than PME18 in the proteomic studies, we hypothesized that disruption of this gene may significantly influence gall anatomy. Previously *pme18-1* (At1g11580, SALK_067447) knockout line was found to be more susceptible to *Pseudomonas syringae* infection (Bethke et al., 2014). Since the main focus of this work was the plant immunity, no description of mutant morphology was included. The SALK_067447 T-DNA line has insertion in an intronic region of *PME18* and in fact insertions in three additional loci. We have decided to use different, single T-DNA accession harboring insertion in a coding region of PME18 (SALK_076975; Supplementary Figure S1) and called it *pme18-2*. We found that the *pme18-2* mutant exhibited a substantially altered phenotype even without infection. Plants were smaller than the corresponding wild-type Col-0, and their root systems were more brittle (prone to break during harvesting of the material). Infected *pme18-2* mutant plants had delayed leaf senescence compared to wild type (Figure 3A). The mutation however did not prevent gall formation, the induction of giant cell formation and the development of resting spores (Figures 3B,D and Supplementary Figure S5). On the other hand, decreased potential for expansive growth in the *pme18-2* mutant resulted in the development of significantly smaller galls (on average by 59.8 and 61.6% at 20 DAI and 26 DAI, respectively; Figure 3E) and cell enlargement observed during organ hypertrophy was reduced (by 50.6 and 61.2%; Figure 3F) in relation to corresponding Col-0 controls. Interestingly, the number of hypertrophied cells in developing galls was also smaller in *pme18-2* compared to Col-0 plants (on average, 71.9 and 49.4%; Figure 3G). Wild-type plants exhibited a significant 37.7% relative increase in gall size from 20 DAI to 26 DAI accompanied with enhanced 76% growth of giant cells (Figures 3E,F), whereas *pme18-2* mutant plants showed no significant relative increase in gall size or giant cell size from 20 DAI to 26 DAI indicating a plateau in gall development. Also, the hypocotyl expansion was significantly reduced in *pme18-2* compared with wild-type Col-0 at 20 and 26 DAI (Supplementary Figure S5B), whereas *pme18-2* hypocotyls expanded by an average 0.93 mm at 20 DAI and 1.08 mm at 26 DAI, in Col-0 hypocotyl widths increased by 2.14 mm and by 2.96 mm, respectively. Assessment of transverse sections of hypocotyls (depicted in Figures 3C,D) also demonstrated dissimilarities in the distribution of hypertrophied cells. In Col-0, giant cells are evenly spread across the hypocotyl. In contrast, the *pme18-2* mutant is characterized by a distinct pattern with hypertrophied cells being predominantly located in the peripheral zone of the hypocotyl. Moreover, 26 DAI giant cells in Col-0 are mostly filled with resting spores, while in *pme18-2*, the central part of the gall still comprises a substantial number of giant cells containing plasmodia alongside some spore-bearing cells (Figures 3C,D and Supplementary Figure S5A).

### Pectin Methylation Is Reduced in Clubroot Galls

**Figure 4:**
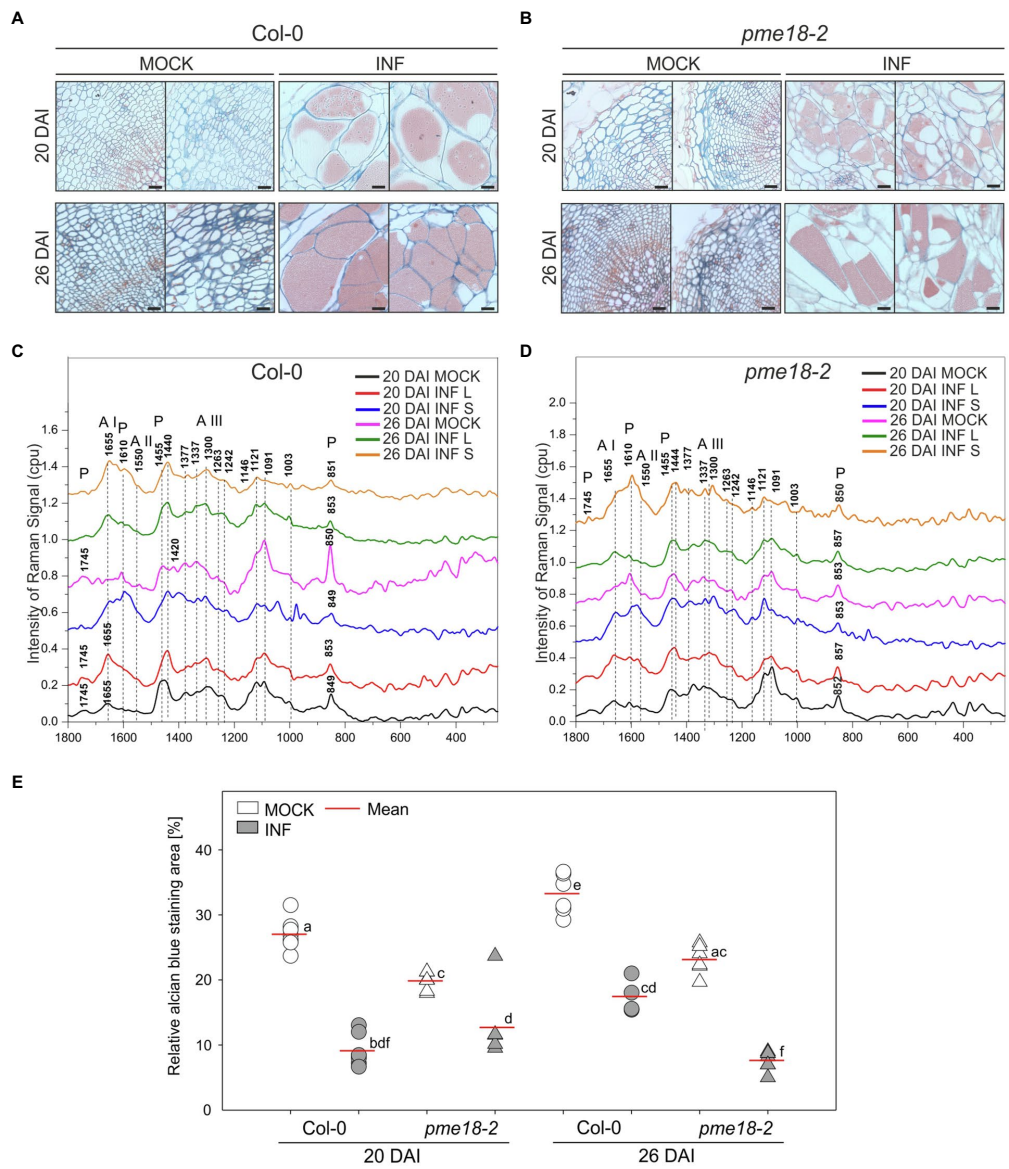
Pectin distribution and pectin methylation status in the hypocotyl cell walls of *P. brassicae*-infected and mock-inoculated Col-0 and *pme18-2* plants. **(A,B)** Fragments of the representative transverse 5 μm sections across hypocotyls of Col-0 and *pme18-2* plants 20 DAI and 26 DAI double-stained with alcian blue (for de-esterified pectin) and safranin O (used as a counterstain). Scale bars represent 100 μm. **(C,D)** Raman spectroscopy results with spectral range of 1800 to 250 cm^−1^ obtained for cell walls in mock-treated and infected hypocotyls of Col-0 and *pme18-2* plants 20 DAI and 26 DAI. Separate measurements for enlarged cells (INF L) and those that did not undergo pathogen-driven expansion (INF S) within galls are presented. The Raman spectra intensities are presented as relative values. To avoid overlapping and facilitate observation of discrete differences spectra were separated. **(E)** Alcian blue staining area in mock- and *P. brassicae*-inoculated Col-0 and *pme18-2* plants 20 DAI and 26 DAI. Scatter plots present individual measurements of alcian blue staining area relative (in %) to the total hypocotyl section area (between four to seven replicates for each combination) and calculated means. Different letters indicate significant differences between means (Tukey’s test, *p* < 0.05).

To gain more insight into the potential role of PME18 in the *P. brassicae*-driven cell expansion, we studied the pectin status of galls. The degree of pectin methylation can be defined as a percentage of carboxyl groups esterified with methanol. De-esterified pectins can be visualized histochemically by alcian blue solution. We found that the intensity of alcian blue staining was slightly lower in Col-0 and *pme18-2* galls compared to representative hypocotyl regions in non-infected controls (Figures 4A,B and E). We followed this observation further and inspected hypocotyl cryo-sections with Raman spectroscopy (Figures 4C,D). This method allowed us to gather information regarding the status of plant cell wall polysaccharides. Combined analysis provides an estimate of the cell wall loosening potential as well as the cellular integrity status in analyzed tissues. Due to the fact that galls are composed of hypertrophied and non-hypertrophied regions, we decided to perform separate scans for enlarged cells (L – large) and for uncolonised cells that did not undergo this process (S – small). Our analysis was based on previous measurements that led to assignment of particular spectra to physico-chemical features of cell wall carbohydrate polymers (Table 1). The spectral patterns obtained are presented in Figures 4C,D, and their characteristics are provided in Table 1. We observed peaks at 1121 cm^−1^ and 1091 cm^−1^ that were previously assigned to the symmetric and asymmetric stretching mode of glycosidic C–O–C bonds in cellulose and hemicelluloses, respectively (Gierlinger et al., 2013). Within the range of 1500 to 1000 cm^−1^, we found other cellulose and hemicellulose bands characteristic mostly for bending and stretching vibration of CH, CH_2_ and OH groups (Himmelsbach and Akin, 1998; Chylińska et al., 2016).

**Table 1:** Assignment of bands in the Raman spectra of cell wall polysaccharides.

Raman wavenumber (cm^−1^; literature)	Assignment	Origin
1745	ν(C=O) methyl ester	P
1655	A I	
1610	ν(COO^−^), asymmetric	P
1550	A II	
1405	ν(COO^−^), symmetric	P
1377	δ(HCC), δ(HCO), δ(HOC)	C
1327	δ(CH)	P
1300	A III	
1263	δ(CH), δ(COH)	H
1242	δ(CH)	P
1121	ν(COC) glycosidic, symmetric	C
1098	ν(COC) glycosidic, asymmetric	C
1003	ν(COOH)	P
971	ρ(CH_2_)	C
842–857	(COC) skeletal mode of α-anomers	P

*C, cellulose; P, pectins; H, hemicellulose; A I-III, amid vibration; ν, stretching vibration; and δ, bending vibration*.

The amount of pectin and degree of methylation were determined based on peaks within the range from 1800 to 250 cm^−1^. We observed the characteristic peaks for stretching vibration of methyl-esterified carboxylic groups (~1745 cm^−1^) and for carboxylate (COO^−^) asymmetric (~1607 cm^−1^) and symmetric (1405 cm^−1^) stretching vibrations (Synytsya et al., 2003). These bands are largely related to the degree of esterification of pectic polysaccharides. Furthermore, a band at 1455 cm^−1^ previously found in Raman spectra of methyl α-D-polygalacturonate and rhamnogalacturonan I (Himmelsbach and Akin, 1998) was detected. The most characteristic band for pectin is a band with a peak between 840 and 857 cm^−1^ which can be assigned to the C–O–C antisymmetric stretch of the α-glycosidic bonds in acidic pectin (Sene et al., 1994). Synytsya et al. (2003) found that this vibration is particularly sensitive to changes of methylation degree of pectic polysaccharides; for highly methylated pectin, its maximum is around 842 cm^−1^ and upon demethylation shifts towards higher wavenumbers to reach 857 cm^−1^ for hypomethylated pectin.

The most pronounced differences in Raman spectra were observed between non-infected and infected tissues. In healthy Col-0, 1745 cm^−1^ and 849 cm^−1^ peaks were detected at both time points (20 and 26 DAI) that indicates pectin methylation. In uninfected *pme18-2* mutants, we could also see the 1745 cm^−1^ peak; however, its intensity was lower than the 1610 cm^−1^ peak, which could indicate that the degree of pectin methylation is somewhat lower than in Col-0 samples. For enlarged cells present in the galls of wild-type Col-0 plants, the band characteristic for glycosidic linkage was shifted from 849 to 853 cm^−1^ and the band at 1745 cm^−1^ vanished. Peaks of 849/851 cm^−1^ were also observed for the Col-0 gall regions where small cells, not colonized by the pathogen, were located which suggests systemic effects on cell walls. It is worth mentioning that there was not only shift but also a decrease in the intensity of the glycosidic linkage band, indicating pectin degradation, observed in galls of infected Col-0 plants (Figure 4C). These effects were particularly pronounced 26 DAI and accompanied by a decrease in the intensity of the 1121 cm^−1^ and 1091 cm^−1^ peaks assigned, respectively, to the symmetric and asymmetric stretching mode of C–O–C in the glycosidic bond in cellulose and hemicelluloses. The degree of pectin methylation observed in the non-infected *pme18-2* mutant is lower than in Col-0 (lower intensity of the 1745 cm^−1^ peak and increase at 1610 cm^−1^). Interestingly, in the mutant, we observed different methylation patterns in small and large cells following infection. For large cells, the band characteristic of the stretching vibration of the glycosidic bond occurs at 852 cm^−1^ (20 DAI) and 853 cm^−1^ (26 DAI) for non-infected samples and is shifted to 857 cm^−1^ after infection. Around the small cells in galls, peaks of 853 cm^−1^ (20 DAI) and 850 cm^−1^ (26 DAI) are present, which suggests that pectin does not undergo demethylation in the cells yet to be colonized and the systemic effects observed in Col-0 are absent (Figure 4D).

**Figure 5:**
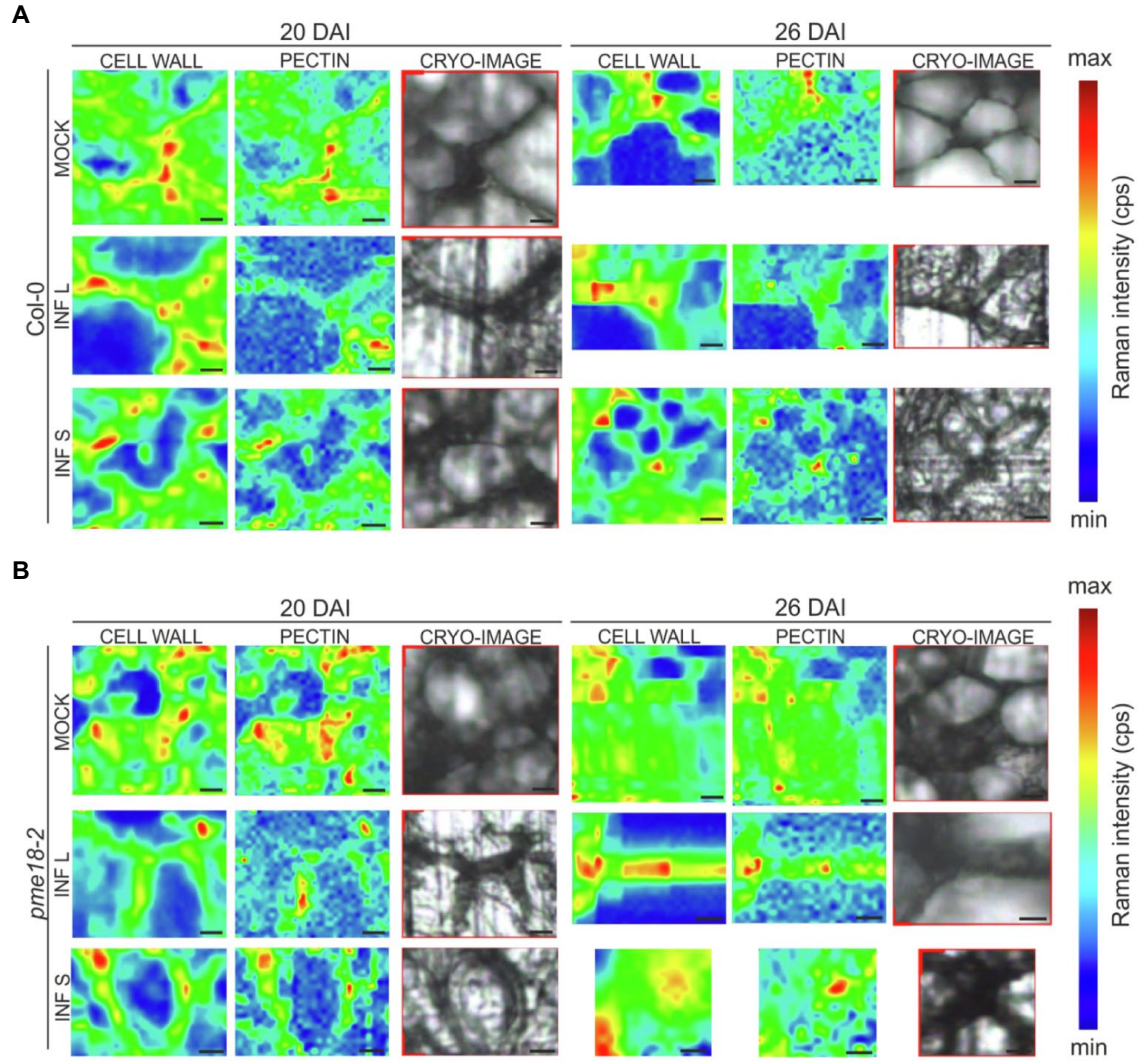
Raman images of cell wall junctions in hypocotyl region of Col-0 plants and the *pme18-2* mutant before and after infection with *P. brassicae*. The Raman maps for cell junction regions of infected (INF) and non-infected (MOCK) cells within hypocotyls were obtained by integrating bands from 2760 to 3100 cm^−1^ (C–H stretching vibration, reflecting distribution of cell wall material) and from 840 to 885 cm^−1^ (corresponds to the band for the (C–O–C) skeletal mode of α-anomers in pectin). On the right side, cryo-section bright field images of scanned regions are provided. Panel **(A)** shows representative cell junctions images for Col-0 genotype, whereas panel **(B)** images for *pme18-2* mutant. Separate images were taken for cell junctions of enlarged cells (INF L) and those that did not undergo pathogen-driven expansion (INF S). Scale bars represent 10 μm. The color scale represents the Raman intensity counts per second of a band visualized in a particular image.

In the obtained spectra, we observed bands characteristic for amide I and III solely in infected samples and this is probably connected with the presence of pathogen (Chisanga et al., 2018). In addition to spectral peak measurements, we studied in detail the cell wall status at cell junctions (Figure 5). Based on obtained Raman images, not only the degree of pectin methylation can be assessed but also its immediate consequences, such as cell wall degradation and loss of cellular integrity. At 20 DAI, both small and enlarged cells within the galls of Col-0 plants maintained relatively well-preserved cell wall structure. At 26 DAI in Col-0, we observed blurred cell edges and a decrease in the glycosidic linkage vibration signal intensity indicating decrease in cell wall integrity (Figure 5A). Distribution maps for samples Col-0 not infected (20 and 26 DAI) clearly presents that pectins were concentrated in cell corners. The pectin distribution pattern observed in the images obtained for *pme18-2* galls (Figure 5B) shows that, despite pectin demethylation, cell walls are better preserved than in wild-type Col-0.

## Discussion and Conclusion

### Proteomic Fingerprint Reflects Host Cell Wall Expansion and Degradation

The application of proteomic methods helps to understand posttranslational aspects of host response regulation. In this study, we were searching for the presence of relatively abundant cell wall proteins that accumulate in cell walls of expanding, pathogen-colonized cells present in hypertrophied hypocotyls. Previous proteomic approaches have focused on the early stages of infection (4 DAI), and a very broad spectrum of changes was analyzed (Devos et al., 2006). In that particular case, total protein extract was analyzed and 46 differentially regulated spots were identified. The main purpose of our work was to identify proteins whose activity during pathogen-driven cell expansion may facilitate cell wall loosening and contribute organ hypertrophy or release of pathogen resting spores to the environment. We decided to perform proteomic studies of the cell wall fraction isolated from healthy hypocotyls and corresponding regions from infected plants during the phase of disease development which is associated with abnormal cell enlargement (20 DAI and 26 DAI). The earlier selected time point represented the state when the highest population of cells was actively expanding, whereas the second (26 DAI) is the time when pathogen resting spore formation occurred inside the majority of colonized cells. Compared to the previous whole proteome studies of Devos et al. (2006), we have identified a limited number of spots exclusively present in cell wall protein extracts from infected tissues. In infected hypocotyls at 20 DAI, we observed accumulation of extracellular proteins whose activity can be directly linked to cell wall remodeling (peroxidases, PME and β-xylosidase). The only protein, whose activity influences cellular growth, showing high accumulation at 26 DAI in infected plants was PME18. Other peptides accumulating at that stage were ubiquitin-related proteins. This change in protein fingerprints may reflect the fact that at 26 DAI, resting spores are formed and numerous processes of protein degradation within the cell (Donald et al., 2008) occur so factors involved in degradation co-extract with the extracellular fraction.

We decided to focus on the finding related to PME18 accumulation. Western blot study has shown a significant increase in the amount of this protein at 20 DAI, but no significant changes were observed at 26 DAI. The discrepancy between proteomics and Western blot results may be related to the fact that at 26 DAI, protein degradation is taking place in galls and some of the detected peptides may originate from partially degraded PME18. Increase in PME18 protein was not reflected at the transcriptional level. The inconsistency between expression of cell wall-related genes and their abundance or activity has been previously discussed and the importance of posttranslational regulation and degradation has been underlined (Vogel and Marcotte, 2012). A proteomic approach, particularly one restricted to the cell wall fractions, is less sensitive than a transcriptomic approach; however, if followed with functional studies, it can be a very useful tool that helps to understand extracellular matrix remodeling that is largely regulated by substrate availability or protein stability (Seifert and Seifert and Blaukopf, 2010). It seems likely that an increase in PME18 protein triggers feedback responses aiming at downregulation of gene expression. This may be a part of trade-off between the maintenance of host cellular integrity and pathogen-driven cell wall degradation. A general overview on the expression of genes whose products are involved in cell wall loosening or degradation during late stages of gall development in Arabidopsis shows that only a limited number of genes are induced and a large number of them are in fact downregulated (Supplementary Figure S4). Recent work of Badstöber et al. (2020) showed that a large number of *PME* gene transcripts increased in mature galls in *P. brassicae*-infected kohlrabi (*Brassica oleracea*). They also found increased expression of other genes whose products may be involved in cell expansion (*XTH* or *EXP*) or degradation (*GH9 cellulases*) processes. In general, in both experimental systems (*B. oleracea* and Arabidopsis), transcriptomic changes depict cell degradation or expansion; however, emerging patterns may differ due to the host response and the speed of disease progression.

### The Importance of Pectin Changes for Gall Development

Invasion of root systems by *P. brassicae* is accompanied by significant cell wall changes, including local cleavage that allows pathogen movement, cell wall thickening and deformation in cells colonized with plasmodia or enormous cell enlargement involving cell wall loosening (Donald et al., 2008). The maintenance of cellular integrity is an important issue within such a dynamically changing cellular environment as the one arising in clubroot galls (Malinowski et al., 2019). We can expect that changes in the pectin fraction will be vital for influencing all of these processes. The pattern of pectin demethylesterification can be influenced by the cellular environment; therefore, sometimes positive effects on cell wall stiffening or loosening may be observed (Bidhendi and Geitmann, 2015). It has been shown that a non-blockwise mode of demethylation decreases the number of Ca^2+^ bridges, exposing this way, demethylated pectin chains to degradation by polygalacturonases (Willats et al., 2001). With alcian blue staining, we observed that the amount of pectin slightly decreases during late stages of gall development. Further inspection with Raman microscopy has shown that this is accompanied with pectin demethylation. Already at 20 DAI in wild-type Col-0 plants, gall formation was accompanied with the characteristic shift in Raman spectra related to the C–O–C antisymmetric stretch vibration of the α-glycosidic bonds in acidic pectin (from 849 cm^−1^ towards 853 cm^−1^). Synytsya et al. (2003) pointed out that this vibration is sensitive to changes in pectic polysaccharide methylation; for highly methylated pectin, its maximum is around 842 cm^−1^ and upon demethylation it shifts towards higher wavenumbers to reach 857 cm^−1^. Another peak correlating with the degree of pectin esterification (1745 cm^−1^) disappears 26 DAI upon infection additionally pointing to pectin demethylation. The intensity of peaks characteristic for the glycosidic linkage in cellulose (1121 cm^−1^) and hemicellulose (1091 cm^−1^) polymers (Gierlinger et al., 2013) decreased in walls of cells within galls. This suggests that cellular degradation processes accompany pectin demethylation in late stages of gall development. This was also visible on Raman images obtained for a 2936 cm^−1^ peak, characteristic of the CH stretching for cell wall polysaccharides (Gierlinger et al., 2012). Cell wall corners on cell junctions imaged at this wavelength appeared to be less focused which suggests polymer degradation (Figure 5B).

Decreasing the host potential for pectin demethylation (*pme18-2*) resulted in smaller galls and less pronounced organ hypertrophy. The *pme18-2* mutation also influenced distribution of the pathogen restricting resting spore formation mainly to outer layers of the cortex (Figure 3B). At present, we do not know whether this is dictated by restricted pathogen movement or a change in root system metabolism. It has been shown that PMEs can be involved in plant defense responses mainly due to the fact that the degree of methyl esterification determines the host cell wall susceptibility to the pathogen-derived enzymes (Lionetti et al., 2012). Particularly, interesting is that pectin-derived elicitors can be produced in response to lignin modification in plant (Gallego-Giraldo et al., 2020). This shows that developmental/physiological response at the cell wall level can be functionally linked to defense or tolerance signaling. Increased accumulation of PME18 protein has been found already 4 DAI in clubroot susceptible plants (Devos et al., 2006), suggesting the possibility of the involvement of this protein in defense responses. However, since we did not observe any signs of resistance in *pme18-2*, we can speculate that at late stages, it plays a mainly structural role during disease progression and gall formation. The role of the PME18 in pathogen movement restriction was not the main subject of our work, but the obtained gall phenotypes encourage further observation with the use of *in planta* tracking techniques.

### Pectin Demethylesterification and Pathogen-Driven Cell Enlargement

**Figure 6:**
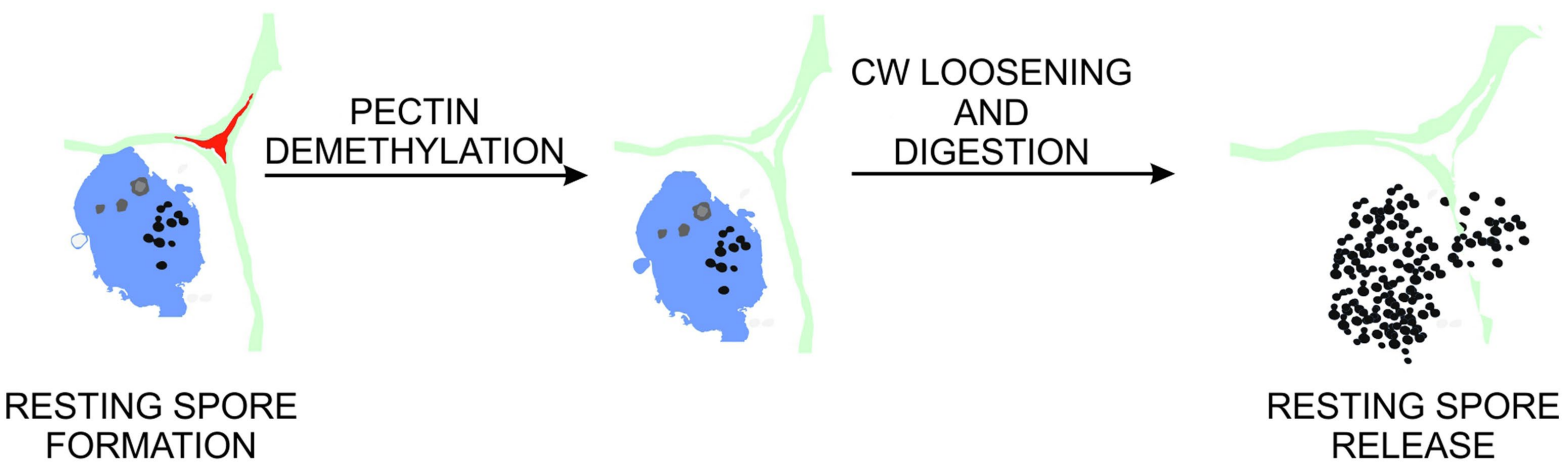
Proposed role of pectin demethylation in gall maturation and resting spore release. At late stages of clubroot disease, cell walls within galls undergo pectin demethylation. This leads to increased exposure of cell wall components to enzymes involved in pectin, cellulose and hemicellulose degradation. These in turn contribute to abnormal hypocotyl hypertrophy and loss of cellular integrity preceding release of pathogen resting spores to the environment.

Based on transcriptomic studies in laser-dissected cells, Schuller et al. (2014) proposed that cell enlargement observed in the host during late stages of disease progression is mediated by auxin-brassinosteroid crosstalk. The exact role of auxin in the activation of PMEs is apparently context dependent since both cell wall loosening and stiffening effects were observed in regions of auxin accumulation. In auxin maxima formed during leaf primordia organogenesis in shoot apex, PME activity leads to a local increase in cell wall extensibility (Peaucelle et al., 2011), whereas during apical hook development auxin co-localized with the region of cellular growth restriction (Jonsson et al., 2021). This shows that other factors can influence the actual effect of pectin demethylation. In our hands, treatment of plants with NPA (a blocker of auxin transport) did not lead to any drastic decrease in cell enlargement taking place during *P. brassicae*-driven hypocotyl hypertrophy (Supplementary Figure S6). This suggests that the enormous growth of cells colonized by the pathogen is triggered locally. At present, we cannot exclude the scenario where factors of pathogen origin mediate these responses; however, based on the work of Schuller et al. (2014), we can say that there is also high possibility that brassinosteroids are involved in the regulation of cellular homeostasis during gall enlargements and some aspects, like cell growth and the maintenance of cellular integrity, are mediated by these phytohormones. Wolf et al. (2012) have shown that the pectin demethylesterification process can be coordinated by the brassinosteroid signaling pathway and such a response is an important component for the maintenance of the cellular integrity during growth. We show that decreased potential for pectin demethylesterification in the *pme18-2* mutant plants results in the reduction of pathogen-driven cell expansion. We cannot exclude however that in some regions of infected hypocotyls, a major role of demethylesterification is in fact the maintenance of cellular integrity during massive tissue outgrowth. Modification of pectin as well as accompanying accumulation of peroxidases observed in later stages of disease progression may also be related to cell wall loosening preceding organ disintegration and release of spores to the environment (Figure 6). It has been shown that demethylesterification of pectin may facilitate cell wall remodeling mediated by peroxidases (Francoz et al., 2015, 2019). It is also intriguing that the *pme18-2* mutation affects not only cell enlargement but also cell proliferation within developing galls – underlining the importance of mechanical coordination of morphogenesis in plant organs. Our finding shows that next to previously described factors, like XTH (Devos et al., 2005) or peroxidases (Ludwig-Müller et al., 1994), PMEs are essential components regulating cellular dynamics during gall formation. In future, we plan to analyze possible feedbacks triggered by the pathogen-driven pectin demethylation in cell walls of hypertrophied hypocotyls. To fully understand the impact of changes in pectin status on *P. brassicae*-driven cell enlargement, more experiments aiming at visualization of changes in associations between particular cell wall polymers and measurements of biophysical properties of their networks should be carried out. Recently, new directions to study the role of pectin in cell enlargement have been proposed by Haas et al. (2021). To rule out the possibility where *pme18-2* has an additional mutation in a different gene that can at least partially contribute to some phenotypic/cellular effects observed in this study, we also plan to include more T-DNA mutants. This is particularly important since so striking effect of a single T-DNA insertion has been observed in an otherwise multigene family.

The decrease in PME18 abundance and resulting lower potential for demethylesterification of pectin does not impede pathogen development; instead, it changes the distribution of resting spore formation. Apparently, space constraints within the gall can only decrease the number of pathogen particles but not progress through its life cycle. This is in agreement with previous experiments where the number of cells within the gall was reduced by overexpression of a negative cell cycle inhibitor KRP1 (Malinowski et al., 2012) yet *P. brassicae* could complete its life cycle. In contrast to that, decreases in nutrient availability for the pathogen *via* reduction of cytokinin biosynthesis (Malinowski et al., 2016) or disruption of carbohydrate delivery (Walerowski et al., 2018) slow down disease progression.

## Data Availability Statement

The original contributions presented in the study are included in the article/Supplementary Material, further inquiries can be directed to the corresponding author/s. The datasets presented in this study can be found in online repositories. The names of the repository/repositories and accession number(s) can be found at ProteomeXchange Consortium PRIDE repository with the accession 537PXD026660 and 10.6019/PXD026660.

## Author Contributions

KS and RM designed the experiments and analyzed the data. KS, PW, MO and AA performed the practical experimental work. MS-C performed Raman spectroscopy analysis. AK performed analysis of 2D GE results. WT performed the bioinformatics analysis. RM wrote the manuscript with contribution of KS and MS-C. AK, WT and AZ revised the manuscript. All authors contributed to the article and approved the submitted version.

## Conflict of Interest

The authors declare that the research was conducted in the absence of any commercial or financial relationships that could be construed as a potential conflict of interest.

## Publisher’s Note

All claims expressed in this article are solely those of the authors and do not necessarily represent those of their affiliated organizations, or those of the publisher, the editors and the reviewers. Any product that may be evaluated in this article, or claim that may be made by its manufacturer, is not guaranteed or endorsed by the publisher.

## Footnotes

^1^ http://arabidopsis.info

^2^ http://signal.salk.edu/tdnaprimers.2.html

^3^ https://primer3plus.com/cgi-bin/dev/primer3plus.cgi

^4^ https://www.ebi.ac.uk/ena
