# Supplementary material for: Plasmodiophora brassicae-Triggered Cell Enlargement and Loss of Cellular Integrity in Root Systems Are Mediated by Pectin Demethylation
Source: Front Plant Sci. 2021 Jul 29;12:711838. doi: 10.3389/fpls.2021.711838 (PMC8359924; doi:10.3389/fpls.2021.711838)
Supplement: Supplementary file 1 [file Table_1.pdf]

Table S1 List of primer sequences

Primers used in testing the SALK line		
Target sequence	Forward primer	Reverse Primer
LBb1.3 = left border of the T-DNA insertion sequence	ATTTTGCCGATTTCGGAAC	-
Genomic primers for *pme18-1* (SALK_076975)	GTCAATGGATCGGATCAGATG	TCCTCCATCGAACTCATCATC
PP2A (At1g13320)	TCCGAGATCACATGTTCCAAA CTC	CCGTATCATGTTCTCCACAACC G
**RT-qPCR primers**		
Target gene	Forward primer	Reverse Primer
PME18 (At1g11580)	CACGCTCTACACTCACACGT	GTGATGTAGCTGTCGCGGTA
TIP41 (At4g34270)	TGAACTGGCTGACAATGGAGTG	CATGAGCTTGGCATGACTCTCAC
UBC9 (At4g27960)	TCCTACTTCATGTAGCGCAGGAC	TCCTCCAGAATAAGGGCTATCCG
PP2A (At1g13320)	TCCGAGATCACATGTTCCAAA CTC	CCGTATCATGTTCTCCACAACC G
