# Supplementary material for: Plasmodiophora brassicae-Triggered Cell Enlargement and Loss of Cellular Integrity in Root Systems Are Mediated by Pectin Demethylation
Source: Front Plant Sci. 2021 Jul 29;12:711838. doi: 10.3389/fpls.2021.711838 (PMC8359924; doi:10.3389/fpls.2021.711838)

Fig. S1. The *pme18-2* T-DNA insertion line

(A) Genomic organization of the *PME18* gene with indicated site of the T-DNA insertion within the gene in the *pme18-2* mutant. Binding positions for primers used for testing are marked with arrows and corresponding primer names.

(B) PCR on genomic DNA of Col-0 and *pme18-2* plants demonstrating homozygosity of the mutant *pme18-2* line. Lanes 1: Primer combination LP$_{pme18\text{-}2}$+RP$_{pme18\text{-}2}$; Lanes 2: Primer combination LBb1.3 + RP$_{pme18\text{-}2}$. M: DNA size marker; NTC: no template control. DNA quality was tested with protein phospatase 2 (*PP2A)* specific primers.

(C) Relative expression of *the PME18* in leaves of *the pme18-2* mutant. Expression analysis was performed by qRT-PCR. Expression levels were calculated relative to the *PME18* expression in Col-0 plants 20 DAI and 26 DAI. Statistical analysis and normalisation has been performed using the REST384 software (Pffafl et al. 2002) with 3 reference genes (*PP2A, TIP41* and *UBC9*); n=3; error bars ± SE; **p<0.01; ***p<0.001.

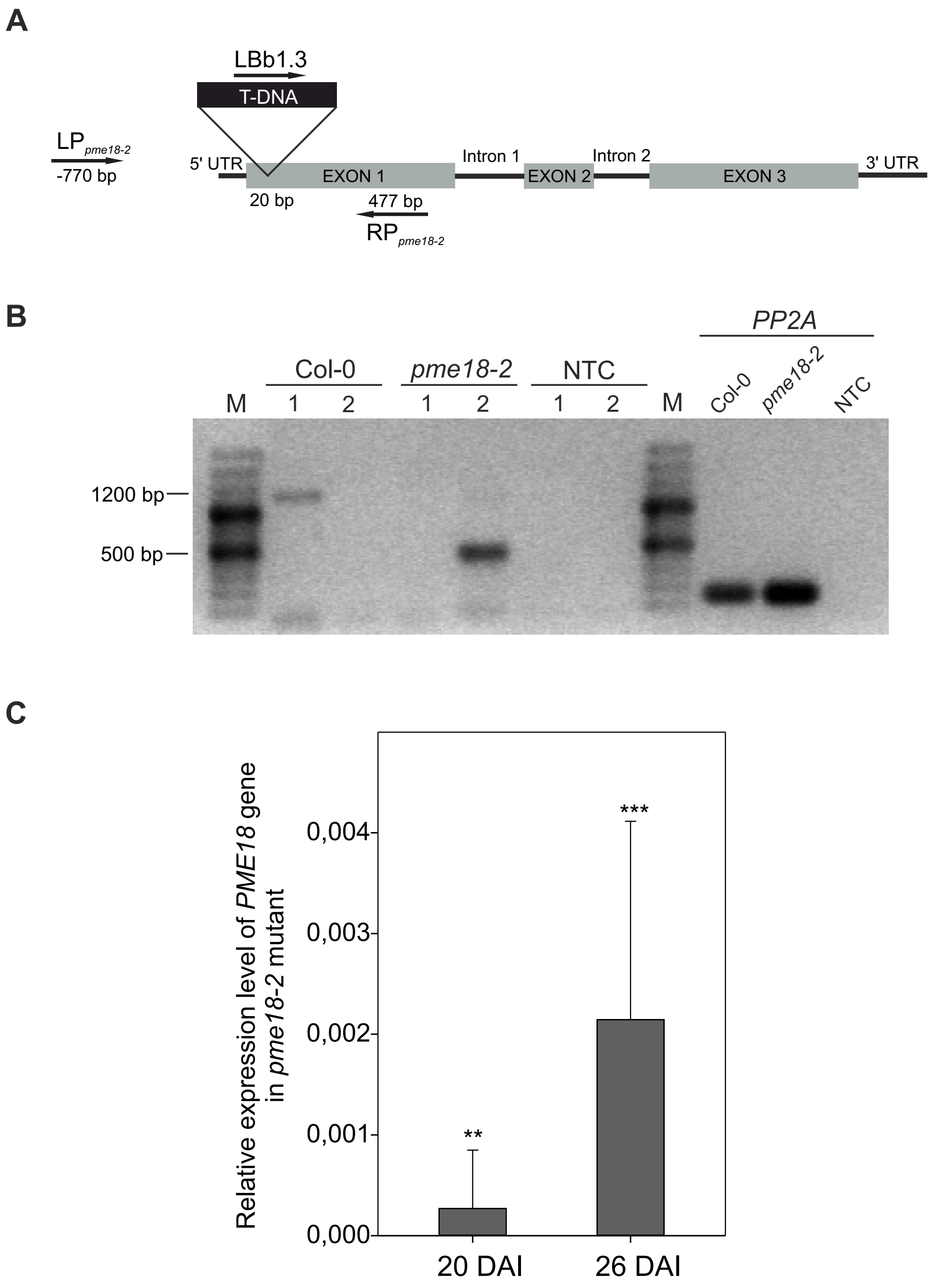

Fig. S2 Representative results of 2-D gel electrophoresis

Raw 2-D protein maps obtained for the cell wall samples of mock and infected Col-0 hypocotyls 20 DAI (A-B), and 26 DAI (C-D). Differential spots (present only in the infected samples) are marked 1-11.

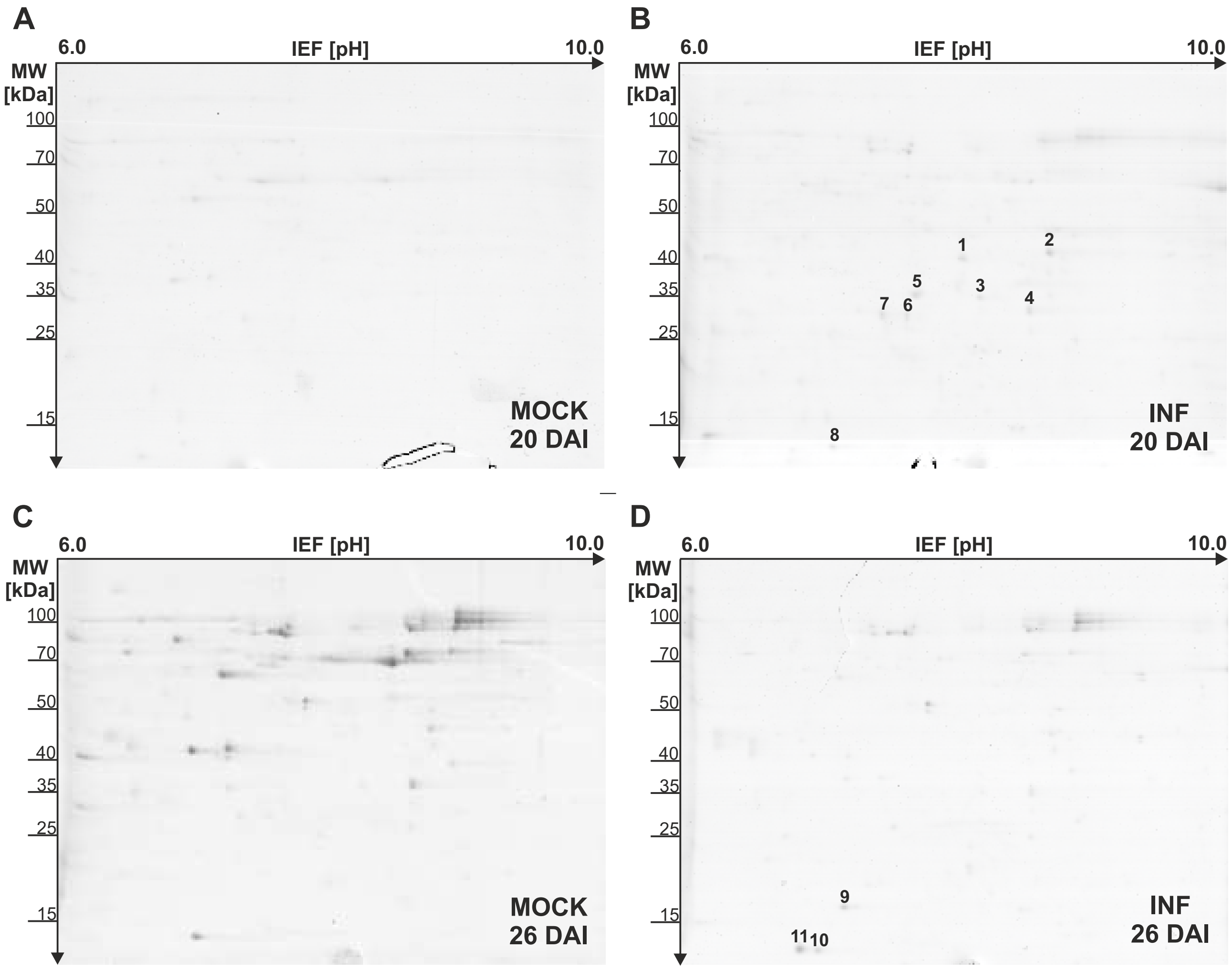

Fig. S3 Amino acid sequences of the identified proteins

Amino acid sequences of peptides which were successfully matched to the proteins are indicated in bold, peptides unique to the identified proteins are underlined. Spot numbers, protein names and database accessions (according to NCBInr) are shown.

Spot 1

Peroxidase 34, CAA50677

1 MHFSSSSTSS TWTILITLGC LMLHASLSAA QLTPTFYDRS CPNVTNIVRE
51 TIVNELRSDP RIAASILRLH FHDCFVNGCD ASILLDNTTS FRTEK**DAFGN**
101 **ANSAR**GFPVI DRMKAAVERA CPRTVSCADM LTIAAQQSVT LAGGPSWRVP
151 LGRRDSLQAF LELANANLPA PFFTLPQLKA SFR**NVGLDRP** **SDLVALSGGH**
201 **TFGKNQCQFI** **LDR**LYNFSNT GLPDPTLNTT YLQTLR**GLCP** **LNGNRSALVD**
251 **FDLRTPTVFD** **NKYYVNLK**ER KGLIQSDQEL FSSPNATDTI PLVR**AYADGT**
301 **QTFFNAFVEA** **MNRMGNITPT** **TGTQGQIR**LN CRVVNSNSLL HDVVDIVDFV
351 SSM

β-xylosidase 7, AAL57631

1 MAKQLLLLLL LFIVHGVESA PPPHSCDPSN PTTKLYQFCR TDLPIGKRAR
51 DLVSRLTIDE KISQLVNTAP GIPRLGVPAY EWWSEALHGV AYAGPGIRFN
101 GTVKAATSFP QVILTAASFD SYEWFRIAQV IGKEARGVYN AGQANGMTFW
151 APNINIFRDP RWGRGQETPG EDPMMTGTYA VAYVRGLQGD SFDGRKTLSN
201 HLQASACCKH FTAYDLDRWK GITRYVFNAQ VSLADLAETY QPPFKKCIEE
251 GRASGIMCAY NRVNGIPSCA DPNLLTRTAR GQWAFRGYIT SDCDAVSIIY
301 DAQGYAKSPE DAVADVLKAG MDVNCGSYLQ KHTKSALQQK KVSETDIDRA
351 LLNLFSVRIR LGLFNGDPTK LPYGNISPNE VCSPAHQALA LDAARNGIVL
401 LKNNLKLLPF SKR**SVSSLAV** **IGPNAHVVK**T LLGNYAGPPC K**TVTPLDALR**
451 SYVKNAVYHQ GCDSVACSNA AIDQAVAIAK **NADHVVLIMG** **LDQTQEK**EDF
501 DR**VDLSLPGK** QQELITSVAN AAKKPVVLVL ICGGPVDISF AANNNKIGSI
551 IWAGYPGEAG GIAISEIIFG DHNPGGRLPV TWYPQSFVNI QMTDMRMR**SA**
601 **TGYPGR**TYKF YKGPKVYEFG HGLSYSAYSY RFKTLAETNL YLNQSKAQTN
651 SDSVRYTLVS EMGKEGCDVA KTKVTVEVEN QGEMAGKHPV LMFARHERGG
701 EDGKRAEKQL VGFKSIVLSN GEKAEMEFEI GLCEHLSRAN EFGVMVLEEG
751 KYFLTVGDSE LPLIVNV

EP1-like glycoprotein 3, AAK96692

1 MKFSITLALC FTLSIFLIGS QAKVPVDDQF RVVNEGGYTD YSPIEYNPDV
51 R**GFVPFSDNF** **R**LCFYNTTPN AYTLALRIGN RVQESTLRWV WEANRGSPVK
101 ENATLTFGED GNLVLAEADG RLVWQTNTAN KGAVGIK**ILE** **NGNMVIYDSS**
151 **GK**FVWQSFDS PTDTLLVGQS LKLNGRTKLV SRLSPSVNTN GPYSLVMEAK
201 KLVLYYTTNK TPKPIAYFEY EFFTKITQFQ SMTFQAVEDS DTTWGLVMEG
251 VDSGSKFNVS TFLSRPKHNA TLSFIRLESD GNIRVWSYST LATSTAWDVT
301 YTAFTNADTD GNDECRIPEH CLGFGLCKKG QCNACPSDKG LLGWDETCKS
351 PSLASCDPKT FHYFKIEGAD SFMTKYNGGS STTESACGDK CTRDCKCLGF
401 FYNRKSSRCW LGYELKTLTR TGDSSLVAYV KAPNANKKST L

Spot 2

Peroxidase 34, CAA50677

1 MHFSSSSTSS TWTILITLGC LMLHASLSAA QLTPTFYDRS CPNVTNIVR**E**
51 **TIVNELR**SDP RIAASILRLH FHDCFVNGCD ASILLDNTTS FRTEK**DAFGN**

101 **ANSAR**GFPVI DRMKAAVERA CPRTVSCADM LTIAAQQSVT LAGGPSWRVP
151 LGRRDSLQAF LELANANLPA PFFTLPQLKA SFR**NVGLDRP SDLVALSGGH**
201 **TFGKNQCQFI LDR**LYNFSNT GLPDPTLNTT YLQTLR**GLCP LNGNRSALVD**
251 **FDLRTPTVFD NKYYVNLK**ER KGLIQSDQEL FSSPNATDTI PLVR**AYADGT**
301 **QTFFNAFVEA MNRMGNITPT TGTQGQIR**LN CRVVNSNSLL HDVVDIVDFV
351 SSM

Peroxidase 33, AAK83646

1 MQFSSSSITS FTWTVLITVG CLMLCASFSD AQLTPTFYDT SCPTVTNIVR
51 DTIVNELRSD PRIAGSILRL HFHDCFVNGC DASILLDNTT SFRTEKDALG
101 NANSARGFPV IDRMKAAVER ACPRTVSCAD MLTIAAQQSV TLAGGPSWKV
151 PLGRRDSLQA FLDLANANLP APFFTLPQLK ANFK**NVGLDR PSDLVALSGA**
201 **HTFGK**NQCRF IMDRLYNFSN TGLPDPTLNT TYLQTLRGQC PRNGNQSVLV
251 DFDLR**TPLVF DNKYYVNLK**E QKGLIQSDQE LFSSPNATDT IPLVR**AYADG**
301 **TQTFFNAFVE AMNRMGNITP TTGTQGQIR**L NCRVVNSNSL LHDVVDIVDF
351 VSSM

Peroxidase 38, CAB78003

1 MHSSLIKLGF LLLLLQVSLS HAQLSPSFYD KTCPQVFDIV TNTIVNALRS
51 DPRIAASILR LHFHDCFVNG CDASILLDNT TSFRTEK**DAF GNANSAR**GFD
101 VIDKMKAAIE KACPRTVSCA DMLAIAAKES IVLAGGPSWM VPNGRRDSLR
151 **GFMDLANDNL PGPSSTLK**QL KDRFKNVGLD R**SSDLVALSG GHTFGKSQCQ**
201 **FIMDRLYNFG ETGLPDPTLD KSYLATLR**KQ CPRNGNQSVL VDFDLR**TPTL**
251 **FDNKYYVNLK** ENKGLIQSDQ ELFSSPDAAD TLPLVRAYAD GQGTFFDAFV
301 KAIIR**MSSLS PLTGK**QGEIR LNCRVVNSKS KIMDVVDDAL EFASFM

β-xylosidase 7, AAL57631

1 MAKQLLLLLL LFIVHGVESA PPPHSCDPSN PTTKLYQFCR TDLPIGKRAR
51 DLVSRLTIDE KISQLVNTAP GIPRLGVPAY EWWSEALHGV AYAGPGIRFN
101 GTVKAATSFP QVILTAASFD SYEWFRIAQV IGKEARGVYN AGQANGMTFW
151 APNINIFRDP RWGRGQETPG EDPMMTGTYA VAYVRGLQGD SFDGRKTLSN
201 HLQASACCK**H FTAYDLDR**WK GITRYVFNAQ VSLADLAETY QPPFKKCIEE
251 GRASGIMCAY NR**VNGIPSCA DPNLLTR**TAR GQWAFRGYIT SDCDAVSIIY
301 DAQGYAK**SPE DAVADVLK**AG MDVNCGSYLQ KHTKSALQQK K**VSETDIDR**A
351 LLNLFSVRIR **LGLFNGDPTK** LPYGNISPNE VCSPAHQALA LDAARNGIVL
401 LKNNLKLLPF SKRSVSSLAV IGPNAHVVKT LLGNYAGPPC KTVTPLDALR
451 SYVKNAVYHQ GCDSVACSNA AIDQAVAIAK NADHVVLIMG LDQTQEKEDF
501 DRVDLSLPGK **QQELITSVAN AAK**KPVVLVL ICGGPVDISF AANNNKIGSI
551 IWAGYPGEAG GIAISEIIFG DHNPGGRLPV TWYPQSFVNI QMTDMRMRSA
601 TGYPGRTYKF YKGPKVYEFG HGLSYSAYSY RFKTLAETNL YLNQSKAQTN
651 SDSVRYTLVS EMGKEGCDVA KTKVTVEVEN QGEMAGKHPV LMFARHERGG
701 EDGKRAEKQL VGFKSIVLSN GEKAEMEFEI GLCEHLSRAN EFGVMVLEEG
751 KYFLTVGDSE LPLIVNV

Eukaryotic aspartyl protease family protein, AAK17160

1 MAPSPIIFSV LLLFIFSLSS SAQTPFRPKA LLLPVTK**DQS TLQYTTVINQ**
51 **RTPLVPASVV FDLGGRELWV DCDKGYVSST YQSPR**CNSAV CSRAGSTSCG
101 TCFSPPRPGC SNNTCGGIPD NTVTGTATSG EFALDVVSIQ STNGSNPGRV
151 VKIPNLIFDC GATFLLKGLA KGTVGMAGMG RHNIGLPSQF AAAFSFHRKF
201 AVCLTSGKGV AFFGNGPYVF LPGIQISSLQ TTPLLINPVS TASAFSQGEK
251 SSEYFIGVTA IQIVEKTVPI NPTLLKINAS TGIGGTKISS VNPYTVLESS

301 IYNAFTSEFV KQAAARSIKR VASVKPFGAC FSTKNVGVTR LGYAVPEIEL
351 VLHSKDVVWR IFGANSMVSV SDDVICLGFV DGGVNARTSV VIGGFQLEDN
401 LIEFDLASNK FGFSSTLLGR QTNCANFNFT STA

Peroxidase 37, AAL40851

1 MHSSLIKLGF LLLLIQVSLS HAQLSPSFYD KTCPQVFDIA TTTIVNALRS
51 DPRIAASILR LHFHDCFVNG CDASILLDNT TSFRTEK**DAF GNANSAR**GFD
101 VIDKMKAAVE KACPKTVSCA DLLAIAAQES VVLAGGPSWR VPNGRRDSLR
151 GFMDLANDNL PAPFFTLNQL KDRFKNVGLD R**ASDLVALSG GHTFGK**NQCQ
201 FIMDRLYNFS NTGLPDPTLD KSYLSTLRKQ CPRNGNQSVL VDFDLR**TPTL**
251 **FDNKYYVNLK** ENKGLIQSDQ ELFSSPDASD TLPLVREYAD GQGKFFDAFA
301 KAMIR**MSSLS PLTGK**QGEIR LNCRVVNSKS KIMDVVEDAL EFASSM

Germin-like protein subfamily 2 member 2, BAD44168

1 MMNSRISIII ALSCIMITSI RAYDPDALQD LCVADKSHGT KLNGFPCKET
51 LNITESDFFF AGISKPAVIN STMGSAVTGA NVEKIPGLNT LSVSLAR**IDY**
101 **APGGLNPPHT HPR**ATEVVYV LEGELEVGFI TTANKLFTKT IKIGEVFVFP
151 R**GLVHFQK**NN GKSPASVLSA FNSQLPGTAS VAATLFAAEP ALPEDVLTKT
201 FQVGSKMVDK IKERLATKK

Germin-like protein subfamily 2 member 1, AAG41457

1 MASPTLTLLL LLTTVSFFIS SSADPDMLQD LCVADLPSGI KINGFPCKDA
51 ATVTSADFFS QGLAKPGLTN NTFGALVTGA NVMTIPGLNT LGVSLSR**IDY**
101 **APGGLNPPHT HPR**ATEVVFV LEGTLDVGFL TTANKLISQS LKKGDVFAFP
151 KGLVHFQKNN GDVPASVIAA FNSQLPGTQS LGATLFGSTP PVPDNILAQA
201 FQTSPGTVKH IKSKFQPKK

Pectin methylesterase 18, AAK59760

1 MSNSNQPLLS KPKSLKHKNL CLVLSFVAIL GSVAFFTAQL ISVNTNNNDD
51 SLLTTSQICH GAHDQDSCQA LLSEFTTLSL SKLNRLDLLH VFLKNSVWRL
101 ESTMTMVSEA RIRSNGVRDK AGFADCEEMM DVSKDRMMSS MEELRGGNYN
151 LESYSNVHTW LSSVLTNYMT CLESISDVSV NSKQIVKPQL EDLVSRARVA
201 LAIFVSVLPA RDDLKMIISN RFPSWLTALD RKLLESSPKT LKVTANVVVA
251 KDGTGKFKTV NEAVAAAPEN SNTRYVIYVK KGVYKETIDI GKKKKNLMLV
301 GDGKDATIIT GSLNVIDGST TFRSATVAAN GDGFMAQDIW FQNTAGPAKH
351 QAVALR**VSAD QTVINR**CR**ID AYQDTLYTHT LR**QFYRDSYI TGTVDFIFGN
401 SAVVFQNCDI VARNPGAGQK **NMLTAQGR**ED QNQNTAISIQ KCK**ITASSDL**
451 **APVK**GSVKTF LGRPWKLYSR TVIMQSFIDN HIDPAGWFPW DGEFALSTLY
501 YGEYANTGPG ADTSKRVNWK GFKVIKDSKE AEQFTVAKLI QGGLWLKPTG
551 VTFQEWL

Spot 3

Leucine-rich repeat (LRR) family protein, AEE31606

1 MNSSFTLFIF TFVIFLQCLN PTGAATCHPD DEAGLLAFK**A GITRDPSGIL**
51 **SSWKKGTACC SWNGVTCLTT DRVSALSVAG QADVAGSFLS GTLSPSLAKL**
101 **KHLDGIYFTD LK**NITGSFPQ FLFQLPNLK**Y VYIENNR**LSG TLPANIGALS
151 QLEAFSLEGN RFTGPIPSSI SNLTLLTQLK LGNNLLTGTI PLGVANLKLM
201 SYLNLGGNRL TGTIPDIFKS MPELRSLTLS RNGFSGNLPP SIASLAPILR

251 **FLELGHNK**LS GTIPNFLSNF KALDTLDLSK NRFSGVIPKS FANLTKIFNL
301 DLSHNLLTDP FPVLNVKGIE SLDLSYNQFH LNTIPKWVTS SPIIFSLKLA
351 KCGIKMSLDD WKPAQTFYYD FIDLSENEIT GSPARFLNQT EYLVEFKAAG
401 NKLRFDMGKL TFAKTLTTLD ISRNLVFGKV PAMVAGLKTL NVSHNHLCGK
451 LPVTKFPASA FVGNDCLCGS PLSPCKA

Eukaryotic aspartyl protease family protein, AAK17160
1 MAPSPIIFSV LLLFIFSLSS SAQTPFRPK**A** **LLLPVTKDQS** **TLQYTTVINQ**
51 **RTPLVPASVV** **FDLGGRELWV** **DCDKGYVSST** **YQSPR**CNSAV CSRAGSTSCG
101 TCFSPPRPGC SNNTCGGIPD NTVTGTATSG EFALDVVSIQ STNGSNPGRV
151 VKIPNLIFDC GATFLLKGLA KGTVGMAGMG RHNIGLPSQF AAAFSFHRKF
201 AVCLTSGKGV AFFGNGPYVF LPGIQISSLQ TTPLLINPVS TASAFSQGEK
251 SSEYFIGVTA IQIVEKTVPI NPTLLKINAS TGIGGTKISS VNPYTVLESS
301 IYNAFTSEFV KQAAARSIKR VASVKPFGAC FSTKNVGVTR LGYAVPEIEL
351 VLHSKDVVWR IFGANSMVSV SDDVICLGFV DGGVNARTSV VIGGFQLEDN
401 LIEFDLASNK FGFSSTLLGR QTNCANFNFT STA

β-xylosidase 7, AAL57631
1 MAKQLLLLLL LFIVHGVESA PPPHSCDPSN PTTKLYQFCR TDLPIGKRAR
51 DLVSRLTIDE KISQLVNTAP GIPRLGVPAY EWWSEALHGV AYAGPGIRFN
101 GTVKAATSFP QVILTAASFD SYEWFRIAQV IGKEARGVYN AGQANGMTFW
151 APNINIFRDP RWGRGQETPG EDPMMTGTYA VAYVR**GLQGD** **SFDGRKTLSN**
201 **HLQASACCKH** **FTAYDLDR**WK GITRYVFNAQ VSLADLAETY QPPFKKCIEE
251 GR**ASGIMCAY** **NRVNGIPSCA** **DPNLLTR**TAR GQWAFRGYIT SDCDAVSIIY
301 DAQGYAK**SPE** **DAVADVLKAG** **MDVNCGSYLQ** **K**HTKSALQQK **KVSETDIDR**A
351 LLNLFSVRIR LGLFNGDPTK LPYGNISPNE VCSPAHQALA LDAARNGIVL
401 LKNNLKLLPF SKRSVSSLAV IGPNAHVVKT LLGNYAGPPC KTVTPLDALR
451 SYVKNAVYHQ GCDSVACSNA AIDQAVAIAK NADHVVLIMG LDQTQEKEDF
501 DRVDLSLPGK QQELITSVAN AAKKPVVLVL ICGGPVDISF AANNNKIGSI
551 IWAGYPGEAG GIAISEIIFG DHNPGGRLPV TWYPQSFVNI QMTDMRMRSA
601 TGYPGRTYKF YKGPKVYEFG HGLSYSAYSY RFKTLAETNL YLNQSKAQTN
651 SDSVRYTLVS EMGKEGCDVA KTKVTVEVEN QGEMAGKHPV LMFARHERGG
701 EDGKRAEKQL VGFKSIVLSN GEKAEMEFEI GLCEHLSRAN EFGVMVLEEG
751 KYFLTVGDSE LPLIVNV

Pectin methylesterase 18, AAK59760
1 MSNSNQPLLS KPKSLKHKNL CLVLSFVAIL GSVAFFTAQL ISVNTNNNDD
51 SLLTTSQICH GAHDQDSCQA LLSEFTTLSL SKLNRLDLLH VFLKNSVWRL
101 ESTMTMVSEA RIRSNGVRDK AGFADCEEMM DVSKDRMMSS MEELRGGNYN
151 LESYSNVHTW LSSVLTNYMT CLESISDVSV NSKQIVKPQL EDLVSRARVA
201 LAIFVSVLPA RDDLKMIISN RFPSWLTALD RKLLESSPKT LKVTANVVVA
251 KDGTGKFKTV NEAVAAAPEN SNTRYVIYVK KGVYKETIDI GKKKKNLMLV
301 GDGKDATIIT GSLNVIDGST TFRSATVAAN GDGFMAQDIW FQNTAGPAK**H**
351 **QAVALRVSAD** **QTVINR**CR**ID** **AYQDTLYTHT** **LR**QFYRDSYI TGTVDFIFGN
401 SAVVFQNCDI VARNPGAGQK **NMLTAQGRED** **QNQNTAISIQ** **K**CK**ITASSDL**
451 **APVKGSVK**TF LGRPWKLYSR TVIMQSFIDN HIDPAGWFPW DGEFALSTLY
501 YGEYANTGPG ADTSKRVNWK GFKVIKDSKE AEQFTVAKLI QGGLWLKPTG
551 VTFQEWL

Eukaryotic aspartyl protease family protein, AAG48774
1 MAPSPIIFSV LLLFIFSLSS SAQTPFRPK**A** **LLLPVTKDPS** **TLQYTTVINQ**

51 **RTPLVPASVV FDLGGR**ELWV DCDKGYVSST YQSPRCNSAV CSRAGSTSCG
101 TCFSPPRPGC SNNTCGGIPD NTVTGTATSG EFALDVVSIQ STNGSNPGRV
151 VKIPNLIFDC GATFLLKGLA KGTVGMAGMG RHNIGLPSQF AAAFSFHRKF
201 AVCLTSGKGV AFFGNGPYVF LPGIQISSLQ TTPLLINPVS TASAFSQGEK
251 SSEYFIGVTA IQIVEKTVPI NPTLLKINAS TGIGGTKISS VNPYTVLESS
301 IYNAFTSEFV KQAAARSIKR VASVKPFGAC FSTKNVGVTR LGYAVPEIEL
351 VLHSKDVVWR IFGANSMVSV SDDVICLGFV DGGVNARTSV VIGGFQLEDN
401 LIEFDLASNK FGFSSTLLGR QTNCANFNFT STA

Serine carboxypeptidase S28 family protein, AAK59466

1 MLSALGFALL SIFAILLSLS TLSNGLLQPR RISHGLTESS KYLTRDELWF
51 NQTLDHYSPS DHREFKQRYY EYLDHLRVPD GPIFMMICGE GPCNGIPNDY
101 ITVLAK**KFDA GIVSLEHR**YY GKSSPFK**SLA TENLK**YLSSK QALFDLAAFR
151 **QYYQDSLNVK** FNRSGDVENP WFFFGASYSG ALSAWFRLKF PHLTCGSLAS
201 SAVVRAVYEF PEFDQQIGES AGPECKAALQ ETNKLLELGL KVNNRAVKAL
251 FNATELDVDA DFLYLIADAE VMAIQYGNPD KLCVPLVEAQ KNRDDLVEAY
301 AKYVREFCVG VFGLSSKTYS RKHLLDTAVT PESADRLWWF QVCTEVAYFQ
351 VAPANDSIRS HQINTEYHLD LCKSLFGKGV YPEVDATNLY YGSDRIAATK
401 IIFTNGSQDP WRHASKQTSS PELPSYIVTC HNCGHGSDLR GCPQSPMVIG
451 GDSKNCSSPD AVNKVRQHIV DHMDLWLSEC RGGIRSSM

Peroxidase 34, CAA50677

1 MHFSSSSTSS TWTILITLGC LMLHASLSAA QLTPTFYDRS CPNVTNIVR**E**
51 **TIVNELR**SDP RIAASILRLH FHDCFVNGCD ASILLDNTTS FRTEK**DAFGN**
101 **ANSAR**GFPVI DRMKAAVERA CPRTVSCADM LTIAAQQSVT LAGGPSWRVP
151 LGRRDSLQAF LELANANLPA PFFTLPQLKA SFR**NVGLDRP SDLVALSGGH**
201 **TFGKNQCQFI LDR**LYNFSNT GLPDPTLNTT YLQTLRGLCP LNGNRSALVD
251 FDLRTPTVFD NKYYVNLKER KGLIQSDQEL FSSPNATDTI PLVRAYADGT
301 QTFFNAFVEA MNR**MGNITPT TGTQGQIR**LN CRVVNSNSLL HDVVDIVDFV
351 SSM

Spot 4

Peroxidase 34, CAA50677

1 MHFSSSSTSS TWTILITLGC LMLHASLSAA QLTPTFYDRS CPNVTNIVR**E**
51 **TIVNELR**SDP R**IAASILR**LH FHDCFVNGCD ASILLDNTTS FRTEK**DAFGN**
101 **ANSARGFPVI DR**MKAAVERA CPR**TVSCADM LTIAAQQSVT LAGGPSWR**VP
151 LGRRDSLQAF LELANANLPA PFFTLPQLKA SFRNVGLDRP SDLVALSGGH
201 TFGKNQCQFI LDRLYNFSNT GLPDPTLNTT YLQTLRGLCP LNGNRSALVD
251 FDLRTPTVFD NKYYVNLKER KGLIQSDQEL FSSPNATDTI PLVRAYADGT
301 QTFFNAFVEA MNR**MGNITPT TGTQGQIR**LN CRVVNSNSLL HDVVDIVDFV
351 SSM

EP1-like glycoprotein 2, AAN60345

1 MSRFAILVTL ALAIATVSVV IAQVPPEKQF RVVNEGEFGE YITEYDASYR
51 FIESSNQSFF TSPFQLLFYN TTPSAYILAL RVGLRRDEST MRWIWDANRN
101 NPVGENATLS LGRNGNLVLA EADGRVKWQT NTANKGVTGF QILPNGNIVL

151 HDKNGKFVWQ SFDHPTDTLL TGQSLKVNGV NKLVSRTSDS NGSDGPYSMV
201 LDKKGLTMYV NKTGTPLVYG GWPDHDFRGT VTFAVTREFD NLTEPSAYEL
251 LLEPAPQPAT NPGNNRRLLQ VRPIGSGGGT LNLNKINYNG TISYLRLGSD
301 GSLK**AYSYFP** **AATYLK**WEES FSFFSTYFVR **QCGLPSFCGD** **YGYCDR**GMCN
351 ACPTPK**GLLG** **WSDK**CAPPKT TQFCSGVKGK **TVNYYKIVGV** **EHFTGPYVND**
401 **GQGPTSVNDC** **K**AKCDRDCK**C** **LGYFYK**EKDK KCLLAPLLGT LIKDANTSSV
451 AYIKY

Eukaryotic aspartyl protease family protein, AAG48774

1 MASSRIIIFS VLLLSIFSLS SSAQPSFRPK **ALLLPVTKDP** **STLQYTTVIN**
51 **QR**TPLVPASV VFDLGGR**EFW** **VDCDQGYVST** **TYR**SPRCNSA VCSRAGSIAC
101 GTCFSPPRPG CSNNTCGAFP DNSITGWATS GEFALDVVSI QSTNGSNPGR
151 FVKIPNLIFS CGSTSLLKGL AKGAVGMAGM GRHNIGLPLQ FAAAFSFNRK
201 FAVCLTSGRG VAFFGNGPYV FLPGIQISRL QKTPLLINPG TTVFEFSKGE
251 KSPEYFIGVT AIKIVEKTLP IDPTLLKINA STGIGGTKIS SVNPYTVLES
301 SIYKAFTSEF IRQAAARSIK RVASVKPFGA CFSTKNVGVT RLGYAVPEIQ
351 LVLHSKDVVW RIFGANSMVS VSDDVICLGF VDGGVNPGAS VVIGGFQLED
401 NLIEFDLASN KFGFSSTLLG RQTNCANFNF TSTA

Peroxidase 37, AAL40851

1 MHSSLIKLGF LLLLIQVSLS HAQLSPSFYD KTCPQVFDIA TTTIVNALRS
51 DPR**IAASILR** LHFHDCFVNG CDASILLDNT TSFRTEK**DAF** **GNANSAR**GFD
101 VIDKMKAAVE KACPKTVSCA DLLAIAAQES VVLAGGPSWR VPNGRRDSLR
151 GFMDLANDNL PAPFFTLNQL KDRFKNVGLD RASDLVALSG GHTFGKNQCQ
201 FIMDRLYNFS NTGLPDPTLD KSYLSTLRKQ CPRNGNQSVL VDFDLR**TPTL**
251 **FDNK**YYVNLK ENKGLIQSDQ ELFSSPDASD TLPLVREYAD GQGKFFDAFA
301 KAMIRMSSLS PLTGKQGEIR LNCRVVNSKS KIMDVVEDAL EFASSM

Leucine-rich repeat (LRR) family protein, AEE31606

1 MNSSFTLFIF TFVIFLQCLN PTGAATCHPD DEAGLLAFKA GITR**DPSGIL**
51 **SSWKKGTACC** **SWNGVTCLTT** **DR**VSALSVAG QADVAGSFLS GTLSPSLAKL
101 K**HLDGIYFTD** **LK**NITGSFPQ FLFQLPNLK**Y** **VYIENNR**LSG TLPANIGALS
151 QLEAFSLEGN RFTGPIPSSI SNLTLLTQLK LGNNLLTGTI PLGVANLKLM
201 SYLNLGGNRL TGTIPDIFKS MPELRSLTLS RNGFSGNLPP SIASLAPILR
251 FLELGHNKLS GTIPNFLSNF KALDTLDLSK NRFSGVIPKS FANLTKIFNL
301 DLSHNLLTDP FPVLNVKGIE SLDLSYNQFH LNTIPKWVTS SPIIFSLKLA
351 KCGIKMSLDD WKPAQTFYYD FIDLSENEIT GSPARFLNQT EYLVEFKAAG
401 NKLRFDMGKL TFAKTLTTLD ISRNLVFGKV PAMVAGLKTL NVSHNHLCGK
451 LPVTKFPASA FVGNDCLCGS PLSPCKA

1 MGFRVCVIVV FLGCLLLVPE KTMAQEMKRA SIVIQGARRV CETDENFVCA
51 TLDWWPHDKC NYDQCPWGYS SVINMDLTRP LLTKAIKAFK PLRIRIGGSL
101 QDQVIYDVGN LKTPCRPFQK MNSGLFGFSK GCLHMKRWDE LNSFLTATGA
151 VVTFGLNALR GRHKLRGKAW GGAWDHINTQ DFLNYTVSKG YVIDSWEFGN
201 ELSGSGVGAS VSAELYGKDL IVLKDVINKV YKNSWLHKPI LVAPGGFYEQ
251 QWYTKLLEIS GPSVVDVVTH HIYNLGSGND PALVKK**IMDP** **SYLSQVSK**TF
301 KDVNQTIQEH GPWASPWVGE SGGAYNSGGR HVSDTFIDSF WYLDQLGMSA
351 RHNTKVYCRQ TLVGGFYGLL EKGTFVPNPD YYSALLWHRL MGK**GVLAVQT**
401 **DGPPQLR**VYA HCSKGRAGVT LLLINLSNQS DFTVSVSNGI NVVLNAESRK
451 KKSLLDTLKR PFSWIGSKAS DGYLNREEYH LTPENGVLRS KTMVLNGKSL

501 KPTATGDIPS LEPVLRSVNS PLNVLPLSMS FIVLPNFDAS ACS

Spot 5

Eukaryotic aspartyl protease family protein, AAK17160

1 MAPSPIIFSV LLLFIFSLSS SAQTPFRPK**A LLLPVTKDQS TLQYTTVINQ**
51 RTPLVPASVV FDLGGRELWV DCDKGYVSST YQSPRCNSAV CSRAGSTSCG
101 TCFSPPRPGC SNNTCGGIPD NTVTGTATSG EFALDVVSIQ STNGSNPGRV
151 V**KIPNLIFDC GATFLLK**GLA KGTVGMAGMG RHNIGLPSQF AAAFSFHRKF
201 AVCLTSGKGV AFFGNGPYVF LPGIQISSLQ TTPLLINPVS TASAFSQGEK
251 SSEYFIGVTA IQIVEKTVPI NPTLLKINAS TGIGGTKISS VNPYTVLESS
301 IYNAFTSEFV KQAAARSIKR VASVKPFGAC FSTKNVGVTR LGYAVPEIEL
351 VLHSKDVVWR IFGANSMVSV SDDVICLGFV DGGVNARTSV VIGGFQLEDN
401 LIEFDLASNK FGFSSTLLGR QTNCANFNFT STA

Eukaryotic aspartyl protease family protein, AAG48774

1 MASSRIIIFS VLLLSIFSLS SSAQPSFRPK **ALLLPVTKDP STLQYTTVIN**
51 **QRTPLVPASV VFDLGGR**EFW VDCDQGYVST TYRSPR**CNSA VCSR**AGSIAC
101 GTCFSPPRPG CSNNTCGAFP DNSITGWATS GEFALDVVSI QSTNGSNPGR
151 FVKIPNLIFS CGSTSLLKGL AKGAVGMAGM GRHNIGLPLQ FAAAFSFNRK
201 FAVCLTSGRG VAFFGNGPYV FLPGIQISRL QKTPLLINPG TTVFEFSKGE
251 KSPEYFIGVT AIKIVEKTLP IDPTLLKINA STGIGGTKIS SVNPYTVLES
301 SIYKAFTSEF IRQAAARSIK RVASVKPFGA CFSTKNVGVT RLGYAVPEIQ
351 LVLHSKDVVW RIFGANSMVS VSDDVICLGF VDGGVNPGAS VVIGGFQLED
401 NLIEFDLASN KFGFSSTLLG RQTNCANFNF TSTA

Leucine-rich repeat (LRR) family protein, AEE31606

1 MNSSFTLFIF TFVIFLQCLN PTGAATCHPD DEAGLLAFKA GITR**DPSGIL**
51 **SSWKKGTACC SWNGVTCLTT DR**VSALSVAG QADVAGSFLS GTLSPSLAK**L**
101 **KHLDGIYFTD LK**NITGSFPQ FLFQLPNLK**Y VYIENNR**LSG TLPANIGALS
151 QLEAFSLEGN RFTGPIPSSI SNLTLLTQLK LGNNLLTGTI PLGVANLKLM
201 SYLNLGGNRL TGTIPDIFKS MPELRSLTLS RNGFSGNLPP SIASLAPILR
251 FLELGHNKLS GTIPNFLSNF KALDTLDLSK NRFSGVIPKS FANLTKIFNL
301 DLSHNLLTDP FPVLNVKGIE SLDLSYNQFH LNTIPKWVTS SPIIFSLKLA
351 KCGIKMSLDD WKPAQTFYYD FIDLSENEIT GSPARFLNQT EYLVEFKAAG
401 NKLRFDMGKL TFAKTLTTLD ISRNLVFGKV PAMVAGLKTL NVSHNHLCGK
451 LPVTKFPASA FVGNDCLCGS PLSPCKA

Peroxidase 34, CAA50677

1 MHFSSSSTSS TWTILITLGC LMLHASLSAA QLTPTFYDRS CPNVTNIVR**E**
51 **TIVNELR**SDP RIAASILRLH FHDCFVNGCD ASILLDNTTS FRTEK**DAFGN**
101 **ANSAR**GFPVI DRMKAAVERA CPRTVSCADM LTIAAQQSVT LAGGPSWRVP
151 LGRRDSLQAF LELANANLPA PFFTLPQLKA SFRNVGLDRP SDLVALSGGH
201 TFGK**NQCQFI LDR**LYNFSNT GLPDPTLNTT YLQTLRGLCP LNGNRSALVD
251 FDLR**TPTVFD NKYYVNLK**ER KGLIQSDQEL FSSPNATDTI PLVRAYADGT
301 QTFFNAFVEA MNR**MGNITPT TGTQGQIR**LN CRVVNSNSLL HDVVDIVDFV
351 SSM

Peroxidase 37, AAL40851

1 MHSSLIKLGF LLLLIQVSLS HAQLSPSFYD KTCPQVFDIA TTTIVNALRS
51 DPRIAASILR LHFHDCFVNG CDASILLDNT TSFRTEK**DAF** **GNANSAR**GFD
101 VIDKMKAAVE KACPKTVSCA DLLAIAAQES VVLAGGPSWR VPNGRRDSLR
151 GFMDLANDNL PAPFFTLNQL KDRFKNVGLD R**ASDLVALSG** **GHTFGKNQCQ**
201 **FIMDR**LYNFS NTGLPDPTLD KSYLSTLRKQ CPRNGNQSVL VDFDLR**TPTL**
251 **FDNKYYVNLK** ENKGLIQSDQ ELFSSPDASD TLPLVREYAD GQGK**FFDAFA**
301 **K**AMIRMSSLS PLTGKQGEIR LNCRVVNSKS KIMDVVEDAL EFASSM

Peroxidase 38, CAB78003

1 MHSSLIKLGF LLLLLQVSLS HAQLSPSFYD KTCPQVFDIV TNTIVNALRS
51 DPRIAASILR LHFHDCFVNG CDASILLDNT TSFRTEK**DAF** **GNANSAR**GFD
101 VIDKMKAAIE KACPR**TVSCA** **DMLAIAAK**ES IVLAGGPSWM VPNGRRDSLR
151 **GFMDLANDNL** **PGPSSTLK**QL KDRFKNVGLD RSSDLVALSG GHTFGKSQCQ
201 FIMDRLYNFG ETGLPDPTLD KSYLATLRKQ CPRNGNQSVL VDFDLR**TPTL**
251 **FDNKYYVNLK** ENKGLIQSDQ ELFSSPDAAD TLPLVRAYAD GQGTFFDAFV
301 KAIIRMSSLS PLTGKQGEIR LNCRVVNSKS KIMDVVDDAL EFASFM

Spot 6

Peroxidase 34, CAA50677

1 MHFSSSSTSS TWTILITLGC LMLHASLSAA QLTPTFYDRS CPNVTNIVR**E**
51 **TIVNELR**SDP R**IAASILR**LH FHDCFVNGCD ASILLDNTTS FRTEK**DAFGN**
101 **ANSARGFPVI** **DR**MKAAVERA CPR**TVSCADM** **LTIAAQQSVT** **LAGGPSWR**VP
151 LGRRDSLQAF LELANANLPA PFFTLPQLKA SFRNVGLDRP SDLVALSGGH
201 TFGKNQCQFI LDRLYNFSNT GLPDPTLNTT YLQTLRGLCP LNGNRSALVD
251 FDLRTPTVFD NK**YYVNLK**ER KGLIQSDQEL FSSPNATDTI PLVRAYADGT
301 QTFFNAFVEA MNR**MGNITPT** **TGTQGQIR**LN CRVVNSNSLL HDVVDIVDFV
351 SSM

Peroxidase 37, AAL40851

1 MHSSLIKLGF LLLLIQVSLS HAQLSPSFYD KTCPQVFDIA TTTIVNALRS
51 DPR**IAASILR** LHFHDCFVNG CDASILLDNT TSFRTEK**DAF** **GNANSARGFD**
101 **VIDK**MKAAVE KACPKTVSCA DLLAIAAQES VVLAGGPSWR VPNGRRDSLR
151 GFMDLANDNL PAPFFTLNQL KDR**FKNVGLD** **RASDLVALSG** **GHTFGKNQCQ**
201 **FIMDR**LYNFS NTGLPDPTLD K**SYLSTLRK**Q CPRNGNQSVL VDFDLR**TPTL**
251 **FDNKYYVNLK** ENK**GLIQSDQ** **ELFSSPDASD** **TLPLVR**EYAD GQGK**FFDAFA**
301 **K**AMIR**MSSLS** **PLTGK**QGEIR LNCRVVNSKS KIMDVVEDAL EFASSM

Peroxidase 38, CAB78003

1 MHSSLIKLGF LLLLLQVSLS HAQLSPSFYD KTCPQVFDIV TNTIVNALRS
51 DPR**IAASILR** LHFHDCFVNG CDASILLDNT TSFRTEK**DAF** **GNANSARGFD**
101 **VIDK**MKAAIE KACPR**TVSCA** **DMLAIAAKES** **IVLAGGPSWM** **VPNGR**RDSLR
151 **GFMDLANDNL** **PGPSSTLK**QL KDR**FKNVGLD** **R**SSDLVALSG GHTFGKSQCQ
201 FIMDRLYNFG ETGLPDPTLD KSYLATLRKQ CPRNGNQSVL VDFDLR**TPTL**
251 **FDNKYYVNLK** ENKGLIQSDQ ELFSSPDAAD TLPLVRAYAD GQGTFFDAFV
301 KAIIR**MSSLS** **PLTGK**QGEIR LNCRVVNSKS KIMDVVDDAL EFASFM

EP1-like glycoprotein 3, AAK96692

1 MKFSITLALC FTLSIFLIGS QAKVPVDDQF R**VVNEGGYTD** **YSPIEYNPDV**

51 **R**GFVPFSDNF RLCFYNTTPN AYTLALRIGN RVQESTLRWV WEANRGSPVK
101 ENATLTFGED GNLVLAEADG RLVWQTNTAN KGAVGIKILE NGNMVIYDSS
151 GKFVWQSFDS PTDTLLVGQS LKLNGRTKLV SRLSPSVNTN GPYSLVMEAK
201 KLVLYYTTNK TPKPIAYFEY EFFTKITQFQ SMTFQAVEDS DTTWGLVMEG
251 VDSGSKFNVS TFLSRPKHNA TLSFIRLESD GNIRVWSYST LATSTAWDVT
301 YTAFTNADTD GNDECR**IPEH CLGFGLCK**KG QCNACPSDKG LLGWDETCK**S**
351 **PSLASCDPK**T FHYFK**IEGDS FMTKYNGGSS TTESACGDK**C TRDCKCLAGF
401 FYNRKSSRCW LGYELKTLTR **TGDSSLVAYV K**APNANKKST L

Leucine-rich repeat (LRR) family protein, AEE31606

1 MNSSFTLFIF TFVIFLQCLN PTGAATCHPD DEAGLLAFKA GITR**DPSGIL**
51 **SSWKKGTACC SWNGVTCLTT DR**VSALSVAG QADVAGSFLS GTLSPSLAKL
101 K**HLDGIYFTD LK**NITGSFPQ FLFQLPNLK**Y VYIENNR**LSG TLPANIGALS
151 QLEAFSLEGN RFTGPIPSSI SNLTLLTQLK LGNNLLTGTI PLGVANLKLM
201 SYLNLGGNRL TGTIPDIFKS MPELRSLTLS RNGFSGNLPP SIASLAPILR
251 FLELGHNKLS GTIPNFLSNF KALDTLDLSK NRFSGVIPKS FANLTKIFNL
301 DLSHNLLTDP FPVLNVKGIE SLDLSYNQFH LNTIPKWVTS SPIIFSLKLA
351 KCGIKMSLDD WKPAQTFYYD FIDLSENEIT GSPARFLNQT EYLVEFKAAG
401 NKLRFDMGKL TFAK**TLTTLD ISR**NLVFGK**V PAMVAGLK**TL NVSHNHLCGK
451 LPVTKFPASA FVGNDCLCGS PLSPCKA

Protein of unknown function, AAL61925

1 MAVPKAIILP ILLLICGAAL GAPASEGYLR NGNFEESPKK TDMKKTVLLG
51 KNALPEWETT GFVEYIAGGP QPGGMYFPVA HGVHAVRLGN EATISQKLEV
101 KPGSLYALTF GASRTCAQDE VLRVSVPSQS GDLPLQTLYN SFGGDVYAWA
151 FVAKTSQVTV TFHNPGVQED PACGPLLDAV AIK**ELVHPIY TR**GNLVKNGG
201 FEEGPHRLVN STQGVLLPPK QEDLTSPLPG WIIESLKAVK FIDSK**YFNVP**
251 **FGHAAIELVA GKESAIAQVI RTSPGQTYTL SFVVGDAKND CHGSMMVEAF**
301 **AARDTLKVPH TSVGGGHVK**T ASFKFKAVEA RTRITFFSGF YHTKKTDTVS
351 LCGPVIDEIV VSHVA

Glyceraldehyde-3-phosphate dehydrogenase GAPC1, AAA32794

1 MADKKIRIGI NGFGRIGRLV ARVVLQRDDV ELVAVNDPFI TTEYMTYMFK
51 YDSVHGQWKH NELKIKDEKT LLFGEKPVTV FGIRNPEDIP WAEAGADYVV
101 ESTGVFTDKD KAAAHLKGGA KKVVISAPSK DAPMFVVGVN EHEYKSDLDI
151 VSNASCTTNC LAPLAKVIND RFGIVEGLMT TVHSITATQK TVDGPSMKDW
201 RGGR**AASFNI IPSSTGAAK**A VGK**VLPALNG K**LTGMSFR**VP TVDVSVVDLT**
251 **VR**LEK**AATYD EIK**KAIKEES EGKLK**GILGY TEDDVVSTDF VGDNR**SSIFD
301 AK**AGIALSDK** FVKLVSWYDN EWGYSSRVVD LIVHMSKA

Spot 7

Peroxidase 34, CAA50677

1 MHFSSSSTSS TWTILITLGC LMLHASLSAA QLTPTFYDRS CPNVTNIVR**E**
51 **TIVNELR**SDP R**IAASILR**LH FHDCFVNGCD ASILLDNTTS FR**TEKDAFGN**
101 **ANSARGFPVI DR**MKAAVERA CPR**TVSCADM LTIAAQQSVT LAGGPSWR**VP
151 LGRRDSLQAF LELANANLPA PFFTLPQLKA SFRNVGLDRP SDLVALSGGH
201 TFGKNQCQFI LDRLYNFSNT GLPDPTLNTT YLQTLRGLCP LNGNRSALVD
251 FDLRTPTVFD NK**YYVNLK**ER KGLIQSDQEL FSSPNATDTI PLVRAYADGT

301 QTFFNAFVEA MNRMGNITPT TGTQGQIRLN CRVVNSNSLL HDVVDIVDFV
351 SSM

Leucine-rich repeat (LRR) family protein, AEE31606

1 MNSSFTLFIF TFVIFLQCLN PTGAATCHPD DEAGLLAFKA GITR**DPSGIL**
51 **SSWKKGTACC** **SWNGVTCLTT** **DRVSALSVAG** **QADVAGSFLS** **GTLSPSLAKL**
101 **KHLDGIYFTD** **LK**NITGSFPQ FLFQLPNLK**Y** **VYIENNRLSG** **TLPANIGALS**
151 **QLEAFSLEGN** **R**FTGPIPSSI SNLTLLTQLK LGNNLLTGTI PLGVANLKLM
201 SYLNLGGNRL TGTIPDIFKS MPELRSLTLS RNGFSGNLPP SIASLAPILR
251 FLELGHNKLS GTIPNFLSNF KALDTLDLSK NRFSGVIPKS FANLTKIFNL
301 DLSHNLLTDP FPVLNVKGIE SLDLSYNQFH LNTIPKWVTS SPIIFSLKLA
351 KCGIKMSLDD WKPAQTFYYD FIDLSENEIT GSPARFLNQT EYLVEFKAAG
401 NKLRFDMGKL TFAKTLTTLD ISRNLVFGK**V** **PAMVAGLK**TL NVSHNHLCGK
451 LPVTKFPASA FVGNDCLCGS PLSPCKA

Glyceraldehyde-3-phosphate dehydrogenase GAPC1, AAA32794

1 MADKKIRIGI NGFGRIGRLV ARVVLQRDDV ELVAVNDPFI TTEYMTYMFK
51 YDSVHGQWKH NELKIKDEKT LLFGEKPVTV FGIRNPEDIP WAEAGADYVV
101 ESTGVFTDKD KAAAHLKGGA KKVVISAPSK DAPMFVVGVN EHEYKSDLDI
151 VSNASCTTNC LAPLAKVIND RFGIVEGLMT TVHSITATQK TVDGPSMKDW
201 RGGRAASFNI IPSSTGAAKA VGK**VLPALNG** **KLTGMSFRVP** **TVDVSVVDLT**
251 **VR**LEK**AATYD** **EIKK**AIKEES EGKLK**GILGY** **TEDDVVSTDF** **VGDNRSSIFD**
301 **AKAGIALSDK** FVKLVSWYDN EWGYSSRVVD LIVHMSKA

Glyceraldehyde-3-phosphate dehydrogenase GAPC2, AAK95257

1 MADKKIRIGI NGFGRIGRLV ARVVLQRDDV ELVAVNDPFI TTEYMTYMFK
51 YDSVHGQWKH HELKVKDDKT LLFGEKPVTV FGIRNPEDIP WGEAGADFVV
101 ESTGVFTDKD KAAAHLKGGA KKVVISAPSK DAPMFVVGVN EHEYKSDLDI
151 VSNASCTTNC LAPLAKVIND RFGIVEGLMT TVHSITATQK TVDGPSMKDW
201 RGGRAASFNI IPSSTGAAKA VGK**VLPSLNG** **KLTGMSFRVP** **TVDVSVVDLT**
251 **VR**LEK**AATYD** **EIKK**AIKEES EGKMK**GILGY** **TEDDVVSTDF** **VGDNRSSIFD**
301 **AKAGIALSDK** FVKLVSWYDN EWGYSSRVVD LIVHMSKA

Peroxidase 38, CAB78003

1 MHSSLIKLGF LLLLLQVSLS HAQLSPSFYD KTCPQVFDIV TNTIVNALRS
51 DPR**IAASILR** LHFHDCFVNG CDASILLDNT TSFR**TEKDAF** **GNANSARGFD**
101 **VIDK**MKAAIE KACPR**TVSCA** **DMLAIAAK**ES IVLAGGPSWM VPNGRRDSLR
151 **GFMDLANDNL** **PGPSSTLK**QL KDRFKNVGLD RSSDLVALSG GHTFGKSQCQ
201 FIMDRLYNFG ETGLPDPTLD KSYLATLRKQ CPRNGNQSVL VDFDLRTPTL
251 FDNK**YYVNLK** ENKGLIQSDQ ELFSSPDAAD TLPLVRAYAD GQGTFFDAFV
301 KAIIRMSSLS PLTGKQGEIR LNCRVVNSKS KIMDVVDDAL EFASFM

EP1-like glycoprotein 3, AAK96692

1 MKFSITLALC FTLSIFLIGS QAKVPVDDQF RVVNEGGYTD YSPIEYNPDV
51 RGFVPFSDNF RLCFYNTTPN AYTLALRIGN R**VQESTLR**WV WEANRGSPVK
101 ENATLTFGED GNLVLAEADG RLVWQTNTAN KGAVGIKILE NGNMVIYDSS
151 GKFVWQSFDS PTDTLLVGQS LKLNGRTKLV SRLSPSVNTN GPYSLVMEAK
201 K**LVLYYTTNK** TPKPIAYFEY EFFTKITQFQ SMTFQAVEDS DTTWGLVMEG
251 VDSGSKFNVS TFLSRPKHNA TLSFIR**LESD** **GNIR**VWSYST LATSTAWDVT
301 YTAFTNADTD GNDECRIPEH CLGFGLCKKG QCNACPSDKG LLGWDETCK**S**

351 **PSLASCDPK**T FHYFK**IEGAD SFMTKYNGGS STTESACGDK** CTRDCKCLGF
401 FYNRKSSRCW LGYELKTLTR **TGDSSLVAYV K**APNANKKST L

Peroxidase 37, AAL40851

1 MHSSLIKLGF LLLLIQVSLS HAQLSPSFYD KTCPQVFDIA TTTIVNALRS
51 DPR**IAASILR** LHFHDCFVNG CDASILLDNT TSFR**TEKDAF GNANSARGFD**
101 **VIDK**MKAAVE KACPKTVSCA DLLAIAAQES VVLAGGPSWR VPNGRRDSLR
151 GFMDLANDNL PAPFFTLNQL KDRFKNVGLD R**ASDLVALSG GHTFGK**NQCQ
201 FIMDRLYNFS NTGLPDPTLD KSYLSTLRKQ CPRNGNQSVL VDFDLRTPTL
251 FDNK**YYVNLK** ENKGLIQSDQ ELFSSPDASD TLPLVREYAD GQGKFFDAFA
301 KAMIRMSSLS PLTGKQGEIR LNCRVVNSKS KIMDVVEDAL EFASSM

Spot 8

Nitrogen regulatory protein P-II homolog, AAC78333

1 MAASMTKPIS ITSLGFYSDR KNIAFSDCIS ICSGFRHSRP SCLDLVTKSP
51 SNNSRVLPVV SAQISSDYIP DSKFYKVEAI VRPWRIQQVS SALLKIGIR**G**
101 **VTVSDVRGFG AQGGSTER**HG GSEFSEDKFV AKVKMEIVVK KDQVESVINT
151 IIEGARTGEI GDGK**IFVLPV SDVIR**VRTGE RGEKAEKMTG DMLSPS

Cysteine proteinase inhibitor 4, AAO22603

1 MMMKSLICLS LILLPLVSVV EGLGGGGGLG SRKPIKNVSD PDVVAVAK**YA**
51 **IEEHNK**ESKE KLVFVK**VVEG TTQVVSGTK**Y DLKIAAKDGG GKIKNYEAVV
101 VEKLWLHSKS LESFKAL

Spot 9

Ubiquitin-like domain-containing protein, BAB10195

1 MEDQPINQCS SSTNASEK**TP ESTLELNIK**T LDSR**TYTFQV NKNETVLLFK**
51 **EKIASETGVP VGQQR**LIFRG RVLKDDHPLS EYHLENGHTL HLIVRQPAES
101 APSSGTPSQG ATANDGNNTN GGPSRNGRHV SHSVVLGSFN VGDQTEGIVP
151 DLSRVIGAVL NSFGVSGQLP TNHSTNGTQS SMPSNQSSNA PPGNTSDGEP
201 GIGGQSQATG HSQPRQAFPG VSFQTSMPRV VQIPVTAATT IPIPSFLTPI
251 PDSLDTLMEF INRMEQALSQ NGYQPDTSSA GSGGRPREEL PRNRRGAATP
301 EALSVVLRNA QHLLSGLGVS SLSHIAGRLE QDGSSSDPTL RSQIQTEAVQ
351 VGLAMQHLGA LLLELGRTIL TLRMAPSPEL SYVNAGPAVY ISPSGPNPIM
401 VQPFPHQISP LFTGATVSSN PLTGPVGLGT AQRHINIHIH AGTSGSPMLS
451 SVGNQRSNGE GGQGDRDSNT SSVPAAVPSH STGENVSAGV QPGLGDDVSV
501 AQINARIRDM VNIMQGRDQI PSGIESLERD MSTGHGVATA MPEQPTNIAT
551 TCAPESSSGS LHDLPSERSN SVCQNEKDLG GDLEHPARAK DTSCTTGQSS
601 APSGDATGDA KETNKATPEV ATATPLGLGL GGLDRKKRSK QPKVSGKTED
651 SGTSATLEGV QQSSGTSGQQ LLQSLFSGSS RSDETGLRRG QGSDDRVDVS
701 SAMSQVLESP VLDGLLAGVS RQAGVDSPNM LRNMLQQFTQ NPQIMNTVQQ
751 IAQQVDGQEI ENMMSGGAQG EGGGFDFSRM VQQMMPLVSR AFSQGGPLPH

801 PATIQPDDRQ PSQVNVQSMA QMIEHSDPPE DVFRAMVENA AISQDELVNE
851 LCCDEALSQE YAELLRRDIE GRLKDDQGL

Thioredoxin H2, CAA84612

1 MGGALSTVFG SGEDATAAGT ESEPSRVLKF SSSARWQLHF NEIKESNKLL
51 VVDFSASWCG PCRMIEPAIH AMADK**FNDVD** **FVKLDVDELP** **DVAKEFNVTA**
101 **MPTFVLVK**RG KEIERIIGAK KDELEKKVSK LRA

THO complex subunit 4B, AAM98152

1 MSGGLDMSLD DIIKSNRKPT GSRGRGGIGG GNNTGGRGGS GSNSGPSRRF
51 ANRVGARTAP YSRPIQQQQA HDAMWQNDVF ATDASVAAAF GHHQTAVVGG
101 GSSIETGTK**L** **YISNLDYGVS** **NEDIKELFSE** **VGDLK**RYGIH YDRSGRSKGT
151 AEVVFSR**RGD** **ALAAVKRYNN** **VQLDGK**LMKI EIVGTNLSAP ALPILATAQI
201 PFPTNGILGN FNENFNGNFN GNFNGNFRGR GRGGFMGRPR GGGFGGGNFR
251 GGRGARGRGG RGSGGRGRDE NVSAEDLDAE LDKYHKEAME TS

Spot 10

Ubiquitin-40S ribosomal protein S27a-1, ABD59101

1 **MQIFVK**TLTG K**TITLEVESS** **DTIDNVK**AK**I** **QDKEGIPPDQ** **QR**LIFAGK**QL**
51 **EDGRTLADYN** **IQKESTLHLV** **LR**LRGGAKKR KKKTYTKPKK IKHTHKKVKL
101 AVLQFYKVDG SGKVQRLKKE CPSVSCGPGT FMASHFDRHY CGKCGTTYVF
151 KKADEE

Polyubiquitin 12, AEE33181

1 MQIFLKTLTG KTKVLEVESS DTIDNVKAKI QDIEGIPPDQ HRLIFAGK**QL**
51 **EDGR**TLADYN VQEDSTLHLL LRFRGGMQIF VKTLTGK**TIT** **LEVESSDTID**
101 **NLKAKIQDKE** **GIPPDQQR**LI FAGKQLEDGR **TLADYNIQKE** **STLHLVLR**LR
151 GGMQIFVKTL TGK**TITLEVE** **SSDTIDNVK**A KIQDKEGISP DQQRLI
201 FAGKQHEDGR TLADYNIQKE STLHLVLRLR GGSF

Polyubiquitin 8, AAA68879

1 MTIQIYAKTL TEKTITLDVE TSDSIHNVKA KIQNKEGIPL DQQRLIFAGK
51 QLEDGLTLAD YNIQK**ESTLH** **LVLR**LRGGMQ IFVQTLTGKT ITLEVK**SSDT**
101 **IDNVK**AKIQD KEGILPRQQR LIFAGK**QLED** **GRTLADYNIQ** **K**ESTLHLVLR
151 LCGGMQIFVS TFSGKNFTSD TLTLKVESSD TIENVKAKIQ DREGLRPDHQ
201 RLIFHGEELF TEDNRTLADY GIRNRSTLCL ALRLRGDMYI FVKNLPYNSF
251 TGENFILEVE SSDTIDNVKA KLQDKERIPM DLHRLIFAGK PLEGGRTLAH
301 YNIQKGSTLY LVTRFRCGMQ IFVKTLTRKR INLEVESWDT IENVKAMVQD
351 KEGIQPQPNL QRLIFLGKEL KDGCTLADYS IQKESTLHLV LGMQIFVKLF
401 GGKIITLEVL SSDTIKSVKA KIQDKVGSPP DQQILLFRGG QLQDGRTLGD
451 YNIRNESTLH LFFHIRHGMQ IFVKTFSFSG ETPTCK**TITL** **EVESSDTIDN**
501 **VK**VKIQHKVG IPLDRQRLIF GGRVLVGSRT LLDYNIQKGS TIHQLFLQRG
551 GMQIFIKTLT GKTIILEVES SDTIANVKEK IQVKEGIKPD QQMLIFFGQQ
601 LEDGVTLGDY DIHKKSTLYL VLRLRQRRYD F

Pectin methylesterase 18, AAK59760

1 MSNSNQPLLS KPKSLKHKNL CLVLSFVAIL GSVAFFTAQL ISVNTNNNDD

51 SLLTTSQICH GAHDQDSCQA LLSEFTTLSL SKLNRLDLLH VFLKNSVWRL
101 ESTMTMVSEA RIRSNGVRDK AGFADCEEMM DVSKDRMMSS MEELRGGNYN
151 LESYSNVHTW LSSVLTNYMT CLESISDVSV NSKQIVKPQL EDLVSRARVA
201 LAIFVSVLPA RDDLKMIISN RFPSWLTALD RKLLESSPKT LKVTANVVVA
251 KDGTGKFKTV NEAVAAAPEN SNTRYVIYVK KGVYKETIDI GKKKKNLMLV
301 GDGKDATIIT GSLNVIDGST TFRSATVAAN GDGFMAQDIW FQNTAGPAKH
351 QAVALR**VSAD** **QTVINRCRID** **AYQDTLYTHT** **LR**QFYRDSYI TGTVDFIFGN
401 SAVVFQNCDI VARNPGAGQK NMLTAQGRED QNQNTAISIQ KCKITASSDL
451 APVKGSVKTF LGRPWKLYSR TVIMQSFIDN HIDPAGWFPW DGEFALSTLY
501 YGEYANTGPG ADTSKRVNWK GFKVIKDSKE AEQFTVAKLI QGGLWLKPTG
551 VTFQEWL

Spot 11

Ubiquitin-40S ribosomal protein S27a-1, ABD59101
1 **MQIFVK**TLTG K**TITLEVESS** **DTIDNVK**AK**I** **QDKEGIPPDQ** **QR**LIFAGK**QL**
51 **EDGRTLADYN** **IQKESTLHLV** **LR**LRGGAKKR KKKTYTKPKK IKHTHKKVKL
101 AVLQFYKVDG SGKVQRLKKE CPSVSCGPGT FMASHFDRHY CGKCGTTYVF
151 KKADEE

Ubiquitin receptor RAD23d, BAC76394
1 MKIFVK**TLSG** **SNFEIEVKPA** **DK**VSDVK**TAI** **ETVKGAEYPA** **AK**QMLIHQGK
51 VLKDETTLEE NNVVENSFIV IMLSKTKASP SGASTASAPA PSATQPQTVA
101 TPQVSAPTAS VPVPTSGTAT AAAPATAASV QTDVYGQAAS NLVAGTTLES
151 TVQQILDMGG GSWDRDTVVR ALRAAFNNPE RAVEYLYSGI PAQAEIPPVA
201 QAPATGEQAA NPLAQPQQAA APAAATGGPN ANPLNLFPQG MPAADAGAGA
251 GNLDFLRNSQ QFQALRAMVQ ANPQILQPML QELGKQNPQL VRLIQEHQAD
301 FLRLINEPVE GEENVMEQLE AAMPQAVTVT PEEREAIERL EGMGFDRAMV
351 LEVFFACNKN EELAANYLLD HMHEFEDQ

Pectin acetylesterase 11, AAL15296
1 MTWLKQMWSS ILVLAVVVIG ARAVPITYLE SAVAKGAVCL DGSAPAYHFD
51 KGSGSGVNNW IVHMEGGGWC TDIATCVQRK STMKGSSKLM NKDFGFSGIL
101 GGKQSTNPDF YNWNRIKVR**Y** **CDGSSFTGDI** **EAVDPTHK**LF FRGARVWRAV
151 IDDLMAKGMS NAQNAILSGC SAGALAAILH CDQFKSTLPK TAKVKCVSDA
201 GYFIHGKDIT GGSYIQSYYA KVVATHGSAK SLPASCTSSM KPDLCFFPQY
251 VAKTLQTPLF VINAAFDSWQ IKNVLAPTSV DKSKAWKTCK LDLKKCTAAQ
301 LQTVQGYRDQ VLAALAPVRS ATTNGLFLDS CHAHCQGGSA ATWSGDKGPT
351 VANTKMAKAV GDWFFERSTF QNVDCSSLNC NPTCPAVSTE D

Fig. S4 Changes in the expression of genes encoding cell wall remodelling proteins triggered by P. brassicae infection

Heat maps present differences between gene expression levels in mock and infected hypocotyls and roots at 16 DAI and 26 DAI defining the proliferative and expansive stages of gall development, respectively. Yellow represents upregulation in the infected tissue relative to the controls, while blue indicates downregulation. The colour bar corresponds to log2 ratios. Results were obtained from RNA-seq profiling of three independent biological replicates, cell wall related genes with significant changes in expression in at least one tissue / timepoint were selected (FDR < 0.05).

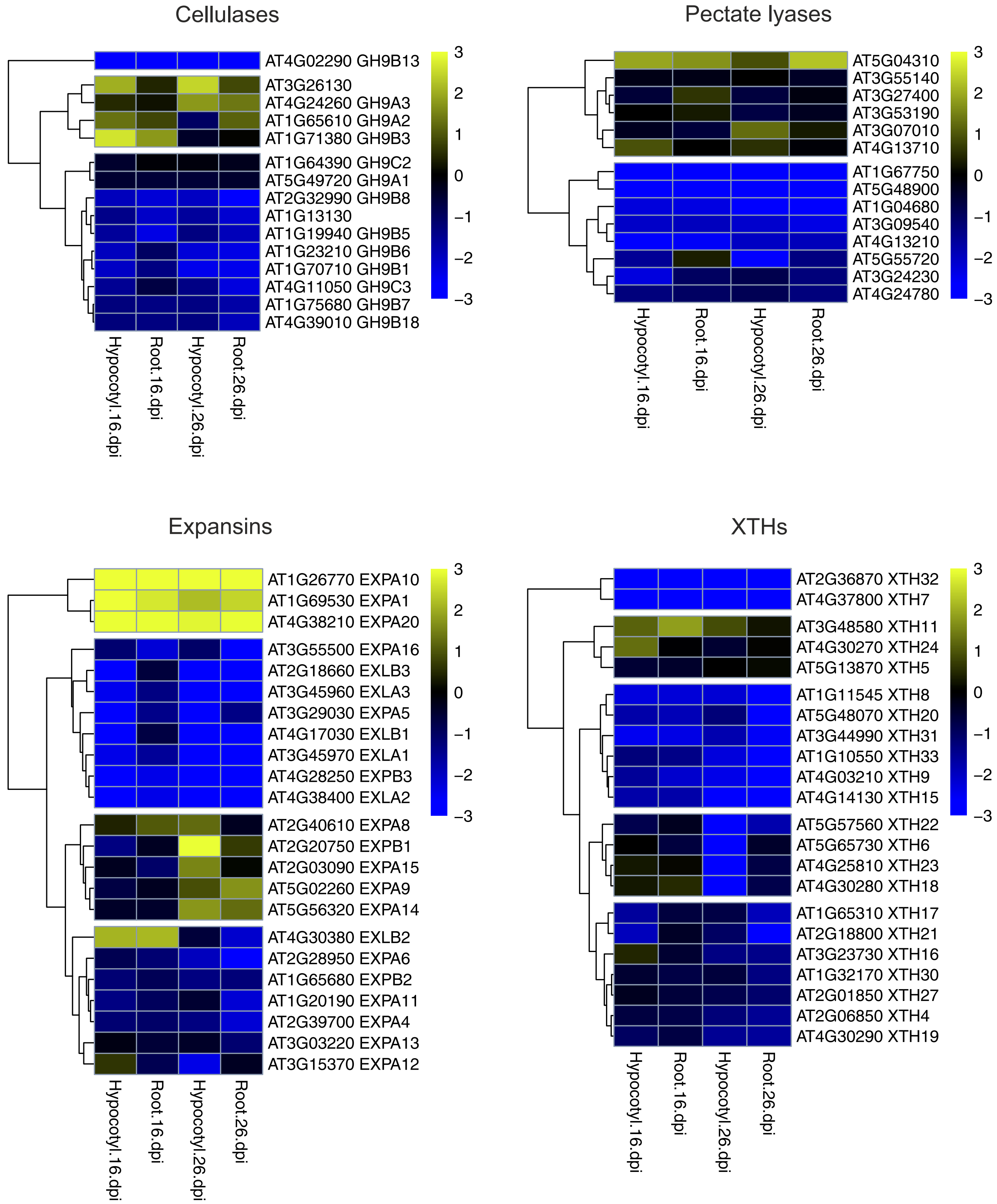

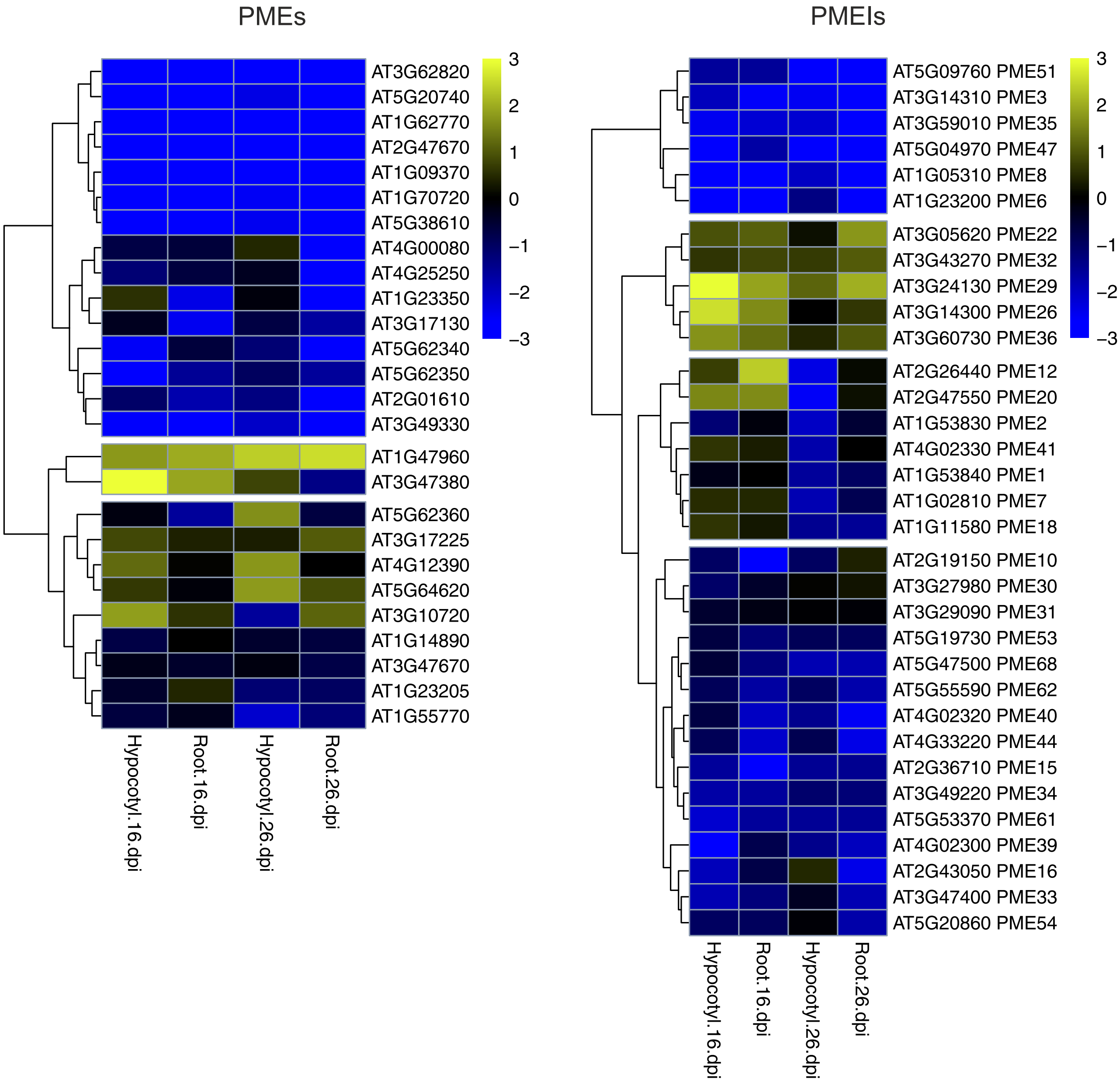
PMEs
AT3G62820
AT5G20740
AT1G62770
AT2G47670
AT1G09370
AT1G70720
AT5G38610
AT4G00080
AT4G25250
AT1G23350
AT3G17130
AT5G62340
AT5G62350
AT2G01610
AT3G49330
AT1G47960
AT3G47380
AT5G62360
AT3G17225
AT4G12390
AT5G64620
AT3G10720
AT1G14890
AT3G47670
AT1G23205
AT1G55770
Hypocotyl.16.dpi
Root.16.dpi
Hypocotyl.26.dpi
Root.26.dpi
3
2
1
0
−1
−2
−3
PMEIs
AT5G09760 PME51
AT3G14310 PME3
AT3G59010 PME35
AT5G04970 PME47
AT1G05310 PME8
AT1G23200 PME6
AT3G05620 PME22
AT3G43270 PME32
AT3G24130 PME29
AT3G14300 PME26
AT3G60730 PME36
AT2G26440 PME12
AT2G47550 PME20
AT1G53830 PME2
AT4G02330 PME41
AT1G53840 PME1
AT1G02810 PME7
AT1G11580 PME18
AT2G19150 PME10
AT3G27980 PME30
AT3G29090 PME31
AT5G19730 PME53
AT5G47500 PME68
AT5G55590 PME62
AT4G02320 PME40
AT4G33220 PME44
AT2G36710 PME15
AT3G49220 PME34
AT5G53370 PME61
AT4G02300 PME39
AT2G43050 PME16
AT3G47400 PME33
AT5G20860 PME54
Hypocotyl.16.dpi
Root.16.dpi
Hypocotyl.26.dpi
Root.26.dpi
3
2
1
0
−1
−2
−3

Fig. S5 *P. brassiacae* disease development in the *pme18-2* mutant in comparison to the wild-type Col-0
(A) Fragments of the representative transverse 5 µm sections across hypocotyls of Col-0 and *pme18-2* plants 20 DAI and 26 DAI stained with toluidine blue. Scale bars represent 100 µm.
(B) Hypocotyl expansion (Δmm width increase) induced by P. brassicae infection calculated from Fig. 3C. Plots represent means ± SE. Different letters indicate significant differences between means (Tamhane's test, P < 0.05).

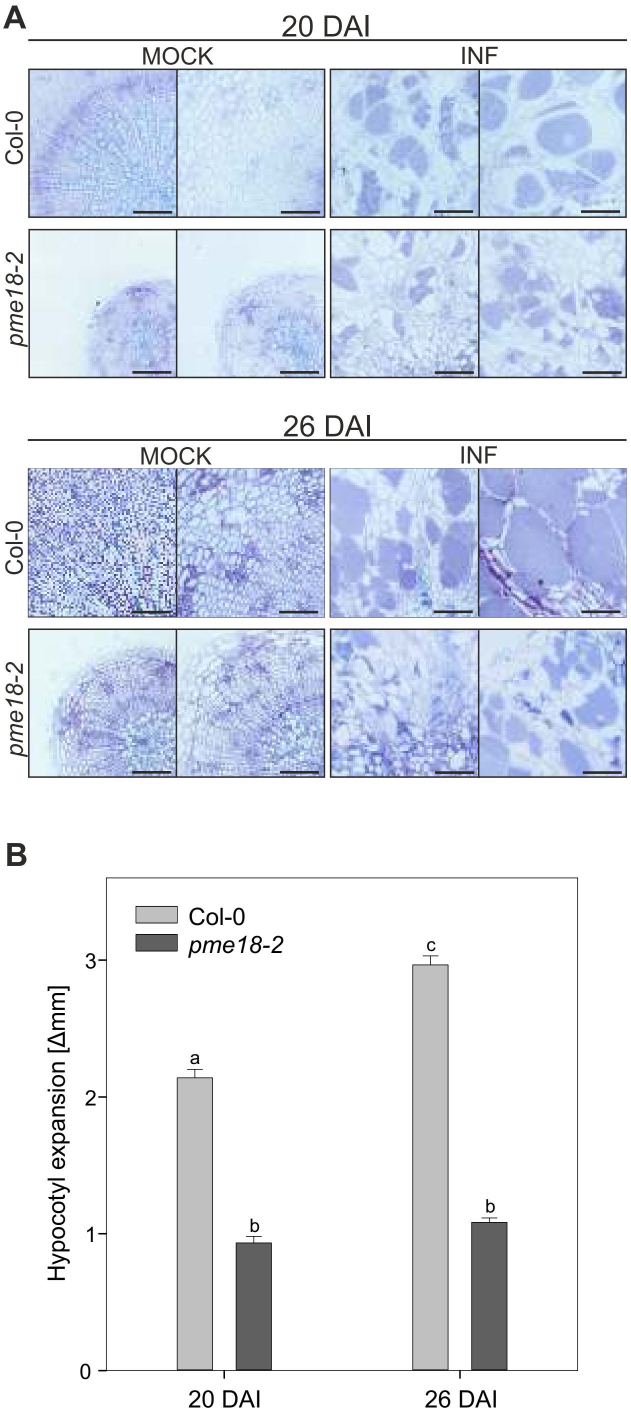

Fig. S6 Effect of NPA treatment on P. brassicae driven cell enlargement
Pictures present cellular changes observed during expansive phase of gall formation visualised 26 DAI in the hypocotyl sections of Col-0 plants untreated and treated with N-1-naphthylphthalamic acid (NPA, auxin transport inhibitor). Scale bars represent 200 μm.

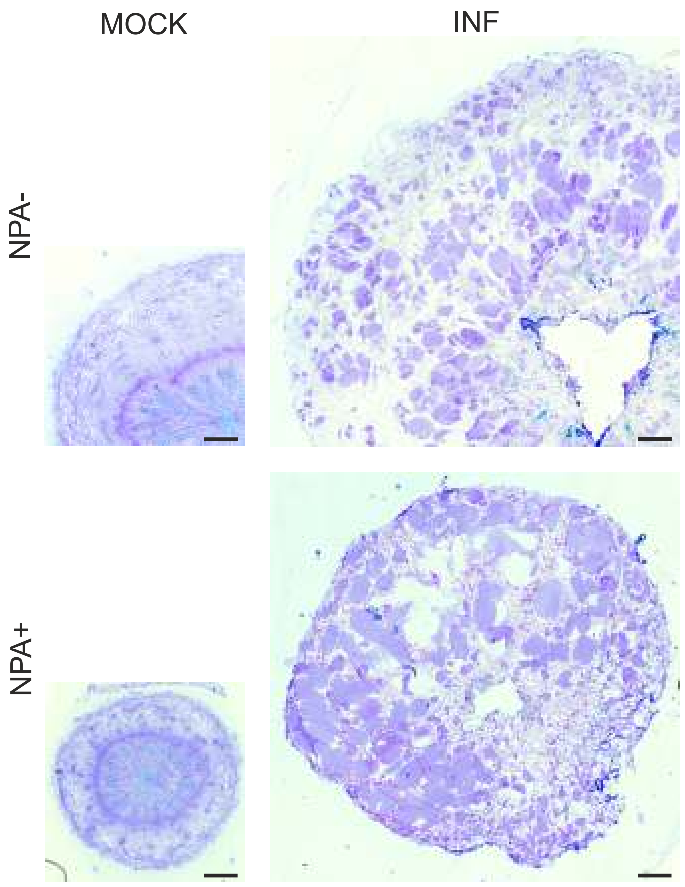

Supplement: Supplementary file 2 [file Data_Sheet_1.PDF]
